# Aspartate β-hydroxylase promotes pancreatic ductal adenocarcinoma metastasis through activation of SRC signaling pathway

**DOI:** 10.1186/s13045-019-0837-z

**Published:** 2019-12-30

**Authors:** Kosuke Ogawa, Qiushi Lin, Le Li, Xuewei Bai, Xuesong Chen, Hua Chen, Rui Kong, Yongwei Wang, Hong Zhu, Fuliang He, Qinggang Xu, Lianxin Liu, Min Li, Songhua Zhang, Katsuya Nagaoka, Rolf Carlson, Howard Safran, Kevin Charpentier, Bei Sun, Jack Wands, Xiaoqun Dong

**Affiliations:** 1 Liver Research Center, Rhode Island Hospital, Warren Alpert Medical School, Brown University, 55 Claverick Street, 4th Fl., Providence, RI 02903 USA; 2 Department of Internal Medicine, College of Medicine, The University of Oklahoma Health Sciences Center, Oklahoma City, OK 731014 USA; 3 Department of Pancreatic and Biliary Surgery; Key Laboratory of Hepatosplenic Surgery, Ministry of Education, The First Affiliated Hospital of Harbin Medical University, Harbin, 150081 Heilongjiang Province People’s Republic of China; 4 Department of Internal Medical Oncology, Harbin Medical University Cancer Hospital, Harbin, 150040 Heilongjiang Province People’s Republic of China; 5 Department of Pathology, The First Affiliated Hospital of Harbin Medical University, Harbin, 150001 Heilongjiang Province People’s Republic of China; 6 Department of Interventional Therapy, Beijing Shijitan Hospital, Capital Medical University, The 9th Affiliated Hospital of Peking University, Beijing, People’s Republic of China; 7 Institute of Life Sciences, Jiangsu University, Zhenjiang, 212013 People’s Republic of China; 8 Department of Hepatic Surgery, Key Laboratory of Hepatosplenic Surgery, Ministry of Education, The First Affiliated Hospital of Harbin Medical University, Harbin, China; 9 Division of Life Sciences and Medicine, The First Affiliated Hospital of USTC, The University of Sciences and Technology of China, No. 17 Lujiang Road, Hefei City, 230001 An Hui Province People’s Republic of China; 10 Immunobiology & Transplant Science Center, Houston Methodist Research Institute, Houston, TX 77030 USA; 11 Division of Hematology/Oncology, Rhode Island Hospital/The Miriam Hospital, The Warren Alpert Medical School of Brown University, Providence, RI USA; 12 Department of Surgery, Rhode Island Hospital, The Warren Alpert Medical School of Brown University, Providence, RI USA; 13 Department of Medicine, The Warren Alpert Medical School of Brown University, Providence, RI USA

**Keywords:** Aspartate β-hydroxylase, Invadopodium, Pancreatic ductal adenocarcinoma, Patient-derived xenograft, Metastasis, SRC

## Abstract

**Background:**

Signaling pathways critical for embryonic development re-emerge in adult pancreas during tumorigenesis. Aspartate β-hydroxylase (ASPH) drives embryonic cell motility/invasion in pancreatic development/differentiation. We explored if dysregulated ASPH is critically involved in pancreatic cancer pathogenesis.

**Methods:**

To demonstrate if/how ASPH mediates malignant phenotypes, proliferation, migration, 2-D/3-D invasion, pancreatosphere formation, immunofluorescence, Western blot, co-immunoprecipitation, invadopodia formation/maturation/function, qRT-PCR, immunohistochemistry (IHC), and self-developed in vitro metastasis assays were performed. Patient-derived xenograft (PDX) models of human pancreatic ductal adenocarcinoma (PDAC) were established to illustrate in vivo antitumor effects of the third-generation small molecule inhibitor specifically against ASPH’s β-hydroxylase activity. Prognostic values of ASPH network components were evaluated with Kaplan-Meier plots, log-rank tests, and Cox proportional hazards regression models.

**Results:**

ASPH renders pancreatic cancer cells more aggressive phenotypes characterized by epithelial–mesenchymal transition (EMT), 2-D/3-D invasion, invadopodia formation/function as demonstrated by extracellular matrix (ECM) degradation, stemness (cancer stem cell marker upregulation and pancreatosphere formation), transendothelial migration (mimicking intravasation/extravasation), and sphere formation (mimicking metastatic colonization/outgrowth at distant sites). Mechanistically, ASPH activates SRC cascade through direct physical interaction with ADAM12/ADAM15 independent of FAK. The ASPH-SRC axis enables invadopodia construction and initiates MMP-mediated ECM degradation/remodeling as executors for invasiveness. Pharmacologic inhibition of invadopodia attenuates in vitro metastasis. ASPH fosters primary tumor development and pulmonary metastasis in PDX models of PDAC, which is blocked by a leading compound specifically against ASPH enzymatic activity. ASPH is silenced in normal pancreas, progressively upregulated from pre-malignant lesions to invasive/advanced stages of PDAC. Expression profiling of ASPH-SRC network components independently/jointly predicts clinical outcome of PDAC patients. Compared to a negative-low level, a moderate-very high level of ASPH, ADAM12, activated SRC, and MMPs correlated with curtailed overall survival (OS) of pancreatic cancer patients (log-rank test, *p*s < 0.001). The more unfavorable molecules patients carry, the more deleterious prognosis is destinated. Patients with 0–2 (*n* = 4), 3–5 (*n* = 8), 6–8 (*n* = 24), and 9–12 (*n* = 73) unfavorable expression scores of the 5 molecules had median survival time of 55.4, 15.9, 9.7, and 5.0 months, respectively (*p* < 0.001).

**Conclusion:**

Targeting the ASPH-SRC axis, which is essential for propagating multi-step PDAC metastasis, may specifically/substantially retard development/progression and thus improve prognosis of PDAC.

**Electronic supplementary material:**

The online version of this article (10.1186/s13045-019-0837-z) contains supplementary material, which is available to authorized users.

## Background

Pancreatic cancer is an intractable malignancy, with 458,918 new cases and 432,242 deaths globally in 2018 [1]. Pancreatic cancer ranks the fourth-leading cause of cancer deaths in the USA, with an estimated of 56,770 new cases and 45,750 deaths in 2019 [2]. Pancreatic cancer has a dismal prognosis; after diagnosis, 25% of patients survive 1 year; only 5–8% survive 5 years. More than 85% of pancreatic cancer is classified as pancreatic ductal adenocarcinoma (PDAC), which is originated from the exocrine glands. Pancreatic cancer is usually symptom-free at the early stage, with gradual progression, and nonspecifically manifests as fatigue, abdominal pain, weight loss, light-colored stools, and jaundice [2]. With disease progression, vast majority of PDAC patients develop relapsed/metastatic tumors invading into the liver, (retro) peritoneal organs, colon, rectum, and lungs. The silent nature, multiple-drug resistance, rapid recurrence, and lack of early detection approaches necessitate active exploration of molecular mechanisms responsible for pancreatic tumorigenesis, progression, and metastasis, so that optimal targets for comprehensive therapy can be identified. We have identified ASPH with potential for achieving this goal. ASPH is a type II transmembrane hydroxylase and a member of highly conserved α-ketoglutarate-dependent hydroxylase (a superfamily non-heme iron-containing protein). The aspartyl and asparaginyl residues in EGF-like repeats of various proteins are natural substrates of ASPH. The embryonic pancreas has been shown to share common signaling pathways with the adult pancreas during malignant transformation. ASPH is a potential driving factor for cell motility and invasion in pancreatic development/differentiation in the embryo [3] and promotes oncogenesis in the adult. ASPH is upregulated by RAS/RAF/MAPK/ERK, PI3K/AKT, and WNT/β-catenin [4–8] to initiate tumorigenesis [4, 9]. However, how ASPH regulates downstream effectors as a determinate for aggressive/invasive phenotypes of pancreatic cancer cells remains mysterious.

Intravasation/extravasation are critical steps for tumor cells to metastasize, however, precise mechanisms regulating these dynamic processes are yet to be clarified. Invadopodia are actin-rich protrusive structures of the plasma membrane to degrade the extracellular matrix (ECM), as a key step for cancer invasion and metastasis. Extending between endothelial cells, intravascular cancer cells “sprout” protrusions [10]. Invadopodia enable the aggressive tumor cells to invade through ECM, intravasate, and extravasate. Invadopodia are composed of structural proteins [e.g., Neural Wiskott-Aldrich syndrome protein (N-WASP), Arp2/3 complex, LIM-kinase, cofilin, cortactin (especially for initiation), Tks5 (important for formation and maturation) and Tks4 (in particular for function) [10]] and ECM-degrading effectors (e.g., MMP14/MT1-MMP, MMP2/9) for locally directed release [11]. Invadopodia can be identified in vivo based on the localization of cortactin, Tks4/Tks5 and MT1-MMP [10]. However, whether invadopodia play a role in ASPH-induced aggressive malignant phenotypes of pancreatic cancer has yet to be disclosed.

## Methods

### Cell lines

Human umbilical vein/vascular endothelium (HUVEC) and pancreatic cancer cell lines were purchased from American type culture collection and authenticated by short tandem repeat profiling to reduce misidentification. HUVECs were grown in complete F-12 K medium and used at passages 5–10. Cancer cells were passaged at 80% of confluence. Stable MIA-Paca2 cell lines overexpressing empty vector and ASPH were established using a lentiviral system (GeneCopoeia, #EX-Z8758-Lv105), whereas stable AsPC-1 and HPAFII cell lines that express CRISPR vector and Cas9-guide RNA (gRNA) specific to ASPH were established using the CRISPR-CAS9 system. All stable cell lines expressing GFP were generated for in vitro metastasis assays.

### Plasmids and reagents

Plasmids Plenti-CMV-ASPH-Lv105 (EX-Z8758-Lv105) and Plenti-CMV-Lv105 empty vector were purchased from GeneCopoeia; pLenti-CMV-GFP-Hygro (656-4) and lentiCRISPR v2 from Addgene (Cambridge, MA); and pRP-Hygro-CMV-ADAM12 and pRP-Hygro-CMV-ADAM15 from Vectorbuild. Dasatinib (CDS023389-25MG) and Wiskostatin (W2270-5MG) were purchased from Sigma-Aldrich and examined at multiple concentrations within an effective range and with minimal off-target effects or toxicity.

### Western Blot

Cell lysates (20–40 μg) were separated by SDS-PAGE and transferred to nitrocellulose membranes using primary antibodies for ASPH (FB50, homemade); MMP14 (#13130S), SRC (#2109S), SRCY416 (#6943S), SRCY527 (#2105S), FAK (#13009S), Phospho-FAK Tyr397 (#8556S), FAK Tyr566/567 (#3281S), FAK Tyr925 (#3284S), and Alexa Fluor® 488 Conjugate (#5198S) from Cell Signaling Technology; MMP1 (sc-58377), ADAM12 (sc-25579), and ADAM15 (sc-16530) from Santa Cruz Biotechnology. Protein bands were visualized by ChemiDoc™ Touch Imaging System (Bio-Rad).

### Migration

Migration was assessed using 24-well Boyden chambers (BD Biosciences). The top chamber (Transwell) with 8.0-μm pores was inserted into a 24-well plate (bottom chamber). 1.2 ml 10% FBS containing medium was added in the bottom chamber as a chemoattractant. Cancer cells (2.5 × 10^4^/well) were seeded onto the top chambers in 500 μl of corresponding serum-free mediums. After incubation for 48–72 h at 37 °C, cancer cells on the top surface were mechanically removed with Q-tips, and migrated cells on the bottom surface were fixed and stained with crystal violet. The average number of migrated cells from 5 to 7 randomly chosen fields on the bottom surface was counted. All data were obtained from ≥ 3 independent experiments.

### 2-D invasion

Cancer cells growing in log phase were incubated in serum-free medium for 24 h. Matrigel invasion chambers (BD BioCoat Matrigel Invasion 24-well Chamber, 8 μm pores, BD Biosciences) were rehydrated for 2 h at 37 °C with corresponding serum-free medium. Immediately prior to the addition of dissociated cancer cells (2.5 × 10^4^/500 μl) to the upper chamber, 750 μl of 10% FBS medium was added to the lower chamber. After incubated for 48–72 h, Matrigel and non-migrating cells were removed from the top chamber with Q-tips. Invading cells on the bottom were fixed in ethanol and stained with crystal violet. After drying overnight, stained cells were counted under a microscope. Percentage of invasion (invasion index) was calculated as a ratio of “Number of invaded cells through Matrigel insert membrane/Number of migrated cells through insert membrane in migration assay”. All data were obtained from ≥ 3 independent experiments.

### Immunoprecipitation

HEK293 cells were cultured in 10-cm dishes and transfected with a total of 10 μg corresponding plasmids when reached 70% of confluence. Cells were harvested using 1 ml PBS containing 1% NP40.

### Pancreatosphere formation

Matrigel (300 μl) was spread evenly to each well of a 24-well plate on ice. The plate was centrifuged at 4 °C, 300×*g*; and immediately placed in a cell culture incubator for 30 min. Single cells were suspended in the medium with 10% Matrigel at a concentration of 2000/400 μl and seeded on Matrigel. Cells were allowed to attach to the Matrigel for 3 h. The medium was carefully removed and replaced with fresh medium containing 10% Matrigel. After incubated for 1 h, corresponding culture medium was added. Fresh medium containing 10% Matrigel was changed every 2 days. The spheres formed after 5–9 days were evaluated in terms of size and number by light microscopy. All experiments were performed in triplicate wells for each condition and repeated in triplicate.

### 3D-embedded and 3D-on top (co-culture layer epi-/endothelial cells on top) cultures

Each well of a 24-well plate was coated with 300 μl of growth factor reduced Matrigel. The plate was incubated at 37 °C under 5% CO_2_ for 30 min. Cancer cells were harvested, counted, and diluted to a concentration of 5000 cells/ml in complete growth medium (containing 2% growth factor reduced Matrigel) of the respective cell line. A total of 400 μl was added to each well of a Matrigel pre-coated plate. The plate was incubated at 37 °C under 5% CO_2_ for 5–7 days. To evaluate the effects of different pharmacologic inhibitors, each compound was added to the complete medium at the time of plating followed by fresh medium changes supplemented with each compound every day while growing the cells on growth factor reduced Matrigel. All experiments were performed in triplicate wells for each condition and repeated in triplicate.

### 3D (spheroid) invasion

To perform 3D Culture 96-Well BME Cell Invasion Assay (Trevigen Inc. Gaithersburg, MD), cancer cell-monolayers were washed with PBS, dissociated by Trypsin, neutralized with complete growth medium. Cells were counted using a hemocytometer. Cell suspension was diluted to 1 × 10^4^ cells/ml (to obtain tumor spheroids of 300–500 μm in diameter 4 days after cell seeding). Cell suspension was dispensed into ULA 96-well round bottom plate and centrifuged at 200×*g* for 5 min. The plate was transferred to an incubator (37 °C, 5% CO_2_, 95% humidity). After 3–5 days, spheroid formation was visually confirmed and proceeded with 3-D invasion assay. Basement membrane matrix was thawed on ice overnight. ULA 96-well plate containing 4-day-old spheroids was placed on ice. In total, 50 μl of basement membrane matrix was gently dispensed into each U-bottom well with six replicates in each group. The plate was centrifuged at 300×*g* for 3 min at 4 °C, then transferred to an incubator at 37 °C, allowing the basement membrane matrix to solidify. After 1 h, 100 μl/well of complete growth medium was gently added into each well. Invasion modulating agents were applied to the system to evaluate its respective impact on cellular phenotype. Spheroid invasion was visualized microscopically and quantitated with NIH IMAGEJ. All experiments were performed in triplicate wells for each condition and repeated in triplicate.

### Invadopodium formation and ECM degradation/remodeling

Cover glass (18 mm; Fisher Scientific) was coated with pig skin green 488 conjugated Gelatin (G13186, Life Technologies). The gelatin was cross-linked with a 0.5% glutaraldehyde solution in a 12-well plate, followed by quenched with sodium borohydride (1 mg/ml) and washed three times with PBS. Pancreatic cancer cells (2 × 10^4^) were seeded to each well in 2 ml of complete medium. After 18–72 h, cells were fixed with 4% paraformaldehyde (PFA), permeabilized with 0.1% Triton X-100, blocked with 5% BSA, and probed for F-actin (Rhodamine phalloidin, R415, Life technologies). The coverslips were mounted over a glass slide with a drop of mounting medium containing DAPI. At least 15 fields per coverslip were imaged at all three channels (red, green, and blue) under × 40 magnification. To quantify invadopodia function, black and white images of gelatin degradation were analyzed using NIH IMAGEJ. The degraded area was normalized to the number of nuclei in the image from the same field. Modulating agents were applied to the system to evaluate its respective impact on cellular phenotype. All experiments were performed in triplicate wells for each condition and performed in triplicate.

### In vitro metastasis

Matrigel invasion chambers (BD BioCoat Matrigel Invasion 24-well Chamber, 8 μm pores, BD Biosciences) were rehydrated for 2 h at 37 °C with serum-free medium. HUVECs (2 × 10^5^) in HUVEC medium were seed in inserted chambers. After 24 h, lower chambers were coated with 290 μl of Matrigel and filled with 500 μl of HUVEC medium containing 10% FBS. Cancer cells (1–4 × 10^4^) stably expressing GFP in HUVEC medium (FBS-free) were plated onto a layer of HUVECs. The plate was incubated in CO_2_ incubator for 3 days. Inserted 24-well chambers were removed, washed with PBS, and fixed with 4% PFA (Sigma-Aldrich) for 20 min, permeabilized with Triton X-100 for 20 min, and stained with phalloidin (red) and Hoest. Transmigrated cancer cells passing through HUVECs were imaged using fluorescence microscope and counted. Cancer cells invaded into the Matrigel within the lower chambers were buried with corresponding medium containing 10% Matrigel, continuously cultured in complete growth medium for 7 days to allow pancreatosphere formation. Tumor spheres were imaged and evaluated in terms of size and number by fluorescence microscope.

### Establishment of PDX model

#### Patient’s tissue procurement

Tumor tissues were collected from six patients (Additional file 7: Table S1) with primary PDAC who had undergone surgical resection. We randomly retrieved 10 surgically resected PDAC specimens from the de-identified archives at the Department of Pathology to illustrate the expression profiling of ASPH network. This study was approved by the Ethics Committee of Institutional Review Board (IRB) at Rhode Island Hospital/Brown University and conducted in accordance with all current ethical guidelines.

### Establishment of F1 generation PDX murine models

PDAC tumor specimens were transferred to the animal procedure room within 0.5–1 h following surgical resection, washed by DMEM supplemented with 1% penicillin/streptomycin, and diced into several fragments (each fragment with an estimated volume of 5 × 5 × 5 mm^3^). Female (5- to 6-week-old; *n* = 6/group) NSG mice (Jackson Laboratory) were kept under pathogen-free conditions, fed standard chow, with free access to sterilized water. These animals were anesthetized with isoflurane (4% induction, 2% maintenance). A small incision was made on the lower back, and one to two tumor fragments were subcutaneously implanted. The residual tumor fragments were formalin-fixed or placed into cryotubes and stored under liquid nitrogen for subsequent immunohistochemistry (IHC) or RT-PCR, respectively. Tumor size was evaluated three times per week by caliper measurements and calculated using the following formula: tumor volume = (length × width^2^)/2. All animal procedures were approved by IACUC at Rhode Island Hospital.

### Establishment of F2 through F7 PDX murine models

When reached approximately 500 mm^3^ in volume, F1 tumors were excised, washed, diced into several fragments (each fragment with an estimated volume of 5 × 5 × 5 mm^3^), and transplanted into 5- to 6-week-old female NSG mice under anesthesia as described for F1 generation mice, and subsequently serially passaged to the F7 generation. Necropsy was performed when the tumors reached approximately 800–900 mm^3^ in volume. The PDX derived tumors, liver and lymph nodes were fixed for routine histology and IHC, and the lungs were placed in Bouin’s solution (HT10132; Sigma-Aldrich, St. Louis, MO) to determine macro-/micro-metastases.

### Antitumor effects of a small molecule inhibitor in vivo

Potential antitumor effects of a small molecule inhibitor [6] on PDAC were analyzed in F5 generation PDX mice. When tumors reached 100 mm^3^ (4–5 weeks after transplantation), mice were randomized into the experimental or control group to be administered with MO-I-1182 or DMSO, respectively. MO-I-1182 (10 mg/kg) was prepared in DMSO and administered by intraperitoneal (IP) injection daily. The oral formulated MO-I-1182 was prepared in capsules at a concentration of 10 mg/kg and administered orally through the esophagus with an injection syringe. At 5 weeks after the initiation of treatment, mice were sacrificed to assess the antitumor effects of i.p. or oral formulated MO-I-1182. Necropsy was performed and primary tumors, lungs, liver, and lymph nodes were surgically removed. The lungs were immersed in Bouin’s solution, and metastatic nodules were counted by visual observation.

### Total RNA extraction and cDNA synthesis

Total RNA was extracted from pancreatic tumor tissue using Trizol reagent (Invitrogen) and then purified with RNeasy mini-spin column (Qiagen). Total RNA (1 μg) of each tumor sample was applied for cDNA synthesis with iScript kit (Bio-Rad) according to the manufacturer’s protocol.

### qRT-PCR

To evaluate a panel of gene expression in pancreatic tumor tissue, synthesized cDNA from prior step was used as a template for qRT-PCR. The assay was performed with gene specific primer sets and a QuantiTect® SYBR® Green PCR kit (Qiagen) on a StepOnePlus Real-Time PCR System (Applied Biosystems). The cycling conditions were as follows: 95 °C for 15 min, then 40 cycles of 94 °C for 15 s, 60 °C for 30 s, and 72 °C for 30 s. Either housekeeping gene GAPDH or β-Actin served as an internal control and the relative mRNA expression was analyzed using 2^−ΔΔCT^ method.

### Expression profile of ASPH network components on human tissue microarray

#### Patient sample collection

The study was approved by the Ethics Committee of IRB at The First Affiliated Hospital of Harbin Medical University, China. All procedures were conducted according to the regulations and guidelines approved by IRB. To quantify ASPH network components in resected tumors from pancreaticoduodenectomy on stage I/II PDAC patients, 166 specimens were randomly selected from banked de-identified tissues without restriction on age, gender, or ethnicity. The tissue bank contains immediately available formalin-fixed paraffin-embedded (FFPE) blocks from PDAC patients as well as complete demographic and clinical characteristics to evaluate the value of ASPH network components expression profiling as a predictor for prognosis.

### Immunohistochemistry (IHC)

Immunohistochemical staining was conducted on 4-μm FFPE unstained sections using primary antibodies for ASPH (FB50, homemade, 1:10,000), MMP1 (ab38929, 1:1000), and MMP14 (ab3644, 1:1000) from Abcam; SRC Y416/418 (PAB25310, 1:100) from Abnova; ADAM12 (14139-1-AP, 1:100) from Proteintech; and reagents from Vector Laboratories (CA, USA). The tissue sections and slides were deparaffinized in xylene and rehydrated in a descending ethanol gradient. Antigen retrieval was performed using citric acid-based antigen unmasking solution in a microwave pressure cooker (Nordic Ware, Minneapolis, MN, USA) for 90 s at full power, followed by cool down for 30 min. Endogenous peroxidase activity was quenched with 3% H_2_O_2_ dissolved in methanol for 30 min. The remaining steps of the staining procedure, including tissue blocking, secondary antibody incubation, and ABC reagent incubation, were performed using VECTASTAIN Elite ABC kit (PK-6101, PK-6102) according to the manufacturer’s instructions. Primary antibodies were diluted in PBS with goat or horse serum attached to ABC kit and were incubated at 4 °C overnight. Color development was performed using a DAB Peroxidase (HRP) substrate kit (SK-4100) as per manufacturer’s instructions. Sections were dehydrated using a reversed ethanol gradient followed by xylene and mounted with Mount-Quick (04970-AB, DAIDO Sango, Tokyo, Japan). Staining intensity and distribution of IHC were assessed by two senior pathologists in a blinded manner at The First Affiliated Hospital of Harbin Medical University. Staining intensity and distribution of IHC were assessed by two senior pathologists. Grade = 0 if intensity = absent, distribution = 0; Grade = 1 if intensity = weak, distribution = 1–25%; Grade = 2 if intensity = moderate, distribution = 26–50%; Grade = 3 if intensity = strong, distribution = 51–75%; Grade = 4 if intensity = very strong, distribution = 75–100%. Cumulative score = staining intensity × staining distribution.

### Statistical analysis

Statistical analyses were performed with SPSS (version 16, IBM) and GraphPad software packages. Nonparametric data (invadopodia) were analyzed with Kruskal-Wallis one-way ANOVA, followed by Tamhane’s post hoc test. Data with normal distributions were represented by mean ± SD and analyzed using one-way ANOVA followed by Bonferroni post hoc. Spearman’s rank correlation coefficient (*ρ*) and Pearson’s correlation coefficient (*r*) were used to quantify the relationship of ASPH expression with other component levels in tumor tissue by IHC. The overall survival (OS) time was calculated from the date of diagnosis to the date of death or last follow-up. Median survival time was estimated using Kaplan-Meier method. Difference in median survival time was examined with log-rank test. Univariate explanatory variables and multivariate Cox proportional hazards regression models were applied to evaluate individual and combined contribution of ASPH network components on OS, adjusting for clinical factors. A *p* < 0.05 (two-tailed) was considered statistically significant.

## Results

### ASPH phenocopies aggressively pathological behaviors, depending on β-hydroxylase activity

Expression profile of ASPH in human pancreatic cancer cell lines has been evaluated previously [12]. The MIA-Paca2 (with a low endogenous level) cell lines stably overexpressing empty vector vs. ASPH were generated using the lentivirus expression system [12]. ASPH was stably knocked out (KO) in AsPC-1 and HPAFII cells (with high endogenous levels) by CRISPR-CAS9 system. Since ASPH’s function depends on β-hydroxylase activity [12], candidate compounds potentially against enzymatic activity of ASPH were developed and screened for bioactivity (Additional file 1: Figure S1A). MO-I-1182 (as the third-generation small molecule inhibitor) has demonstrated a dose-dependent effect on cell viability, which is more potent than the first- (e.g., MO-I-1100) or second-generation (e.g., MO-I-1151) small molecule inhibitors as previously characterized [12].

**Fig. 1:**
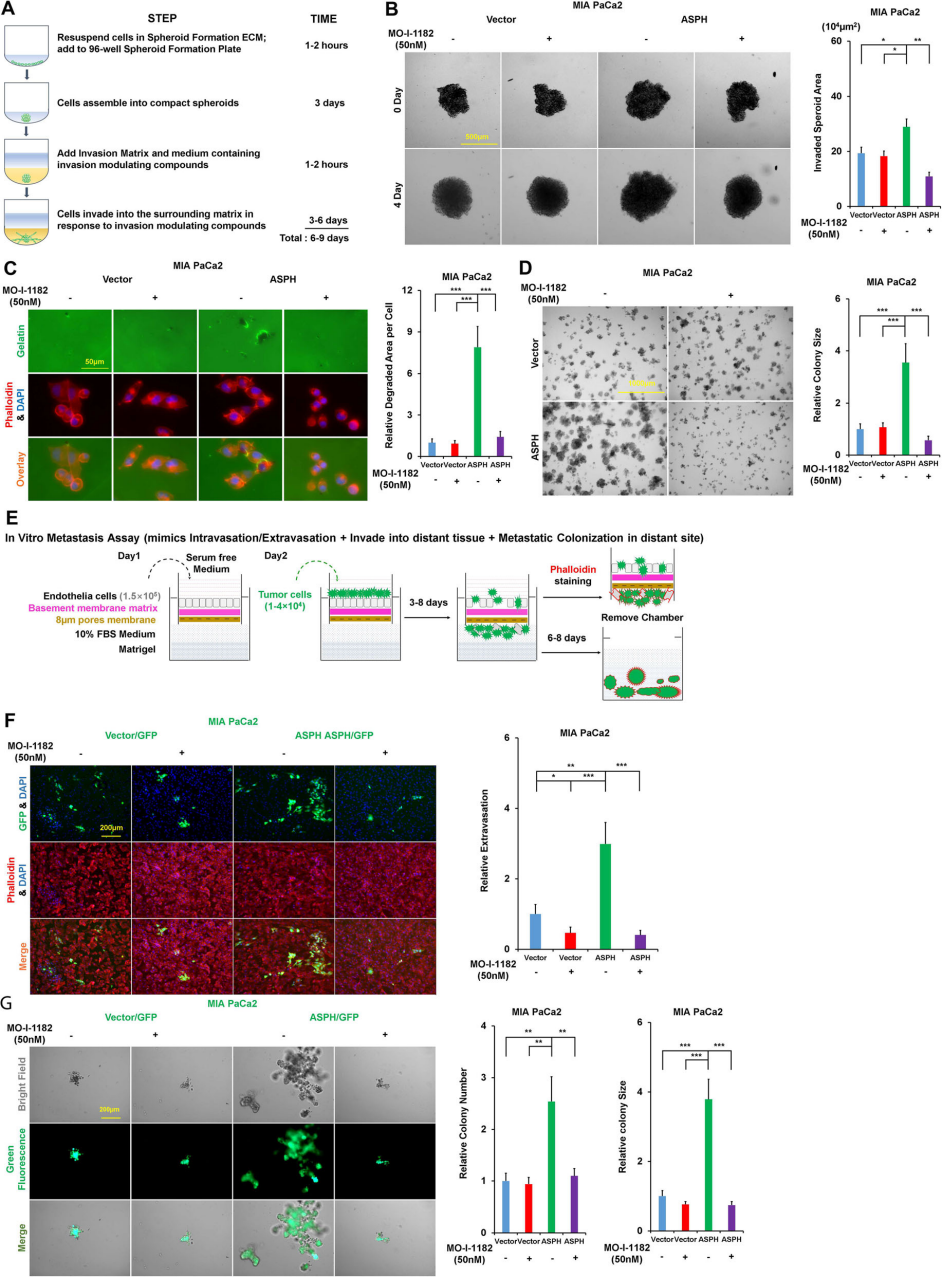
ASPH-mediated aggressive phenotypes are reversed in vitro by a small molecule inhibitor (SMI) specifically against its β-hydroxylase activity in pancreatic cancer cells. **a** Scheme of 3-D tumor spheroid invasion assay. **b** 3-D tumor spheroid invasion in response to SMI. **c** ECM degradation/remodeling in response to SMI. **d** 3-D pancreatosphere formation in response to SMI. **e** Scheme of in vitro metastasis assay of pancreatic cancer cells, which mimics local invasion (penetration through basement membrane) at the primary site, intravasation/extravasation, invasion into distant tissue, and eventual metastatic colonization/outgrowth at distant sites. **f** Transendothelial migration and intravasation/extravasation; **g** Invasion through basement membrane and subsequent pancreatosphere formation in response to SMI. ^*^p < 0.05; ^**^p < 0.01; ^***^p < 0.001

To demonstrate if inhibiting enzymatic activity of ASPH could reverse malignant phenotype of pancreatic cancer cells, serial assays including migration, 2-D invasion, 3D invasion, pancreatosphere formation, and immunofluorescence were performed. In MIA-Paca2, exogenous ASPH substantially spurred migration/2-D invasion (Additional file 1: Figure S1B-C) and epithelial–mesenchymal transition (EMT) as highlighted by downregulated epithelial marker E-cadherin and/or upregulated mesenchymal marker Vimentin (Additional file 1: Figure S1H-I); 3D invasion (Fig. 1a, b), ECM degradation/remodeling (Fig. 1c), and stemness (Fig. 1d; Additional file 2: Figure S2A) as demonstrated by upregulated cancer stem cell markers (CD44 and EpCAM) and/or enhanced pancreatosphere formation. These phenotypes were specifically dismantled by the small molecule inhibitor. Consistently, endogenous ASPH propagated migration/2D invasion (Additional file 1: Figure S1D-G), EMT (Additional file 1: Figure S1J-L), 3D invasion (Additional file 1: Figure S1M), ECM degradation/remodeling (Additional file 1: Figure S1N-O), and stemness (Additional file 2: Figure S2B-I), which were substantially mitigated by the small molecule inhibitor or ASPH KO in AsPC-1 and HPAFII.

To characterize ASPH’s pro-oncogenic properties, in vitro metastasis assay was developed (Fig. 1e). This assay mimics how pancreatic cancer cells invade through the basement membrane at the primary site, subsequently intravasate into/extravasate out of the vasculature system, and consequently form metastatic colonization/outgrowth at distant sites. The ASPH significantly boosted transendothelial migration, subsequent extravasation, and consequent metastatic colonization/outgrowth (pancreatosphere formation) of MIA-Paca2 compared to the empty vector, which were inhibited by MO-I-1182 (Fig. 1f, g). Endogenous ASPH-mediated in vitro metastasis was abolished by MO-I-1182 or ASPH KO in AsPC-1 (Additional file 2: Figure S2J-K).

### ASPH physically interacts with ADAM12/15 to activate SRC cascade

**Fig. 2:**
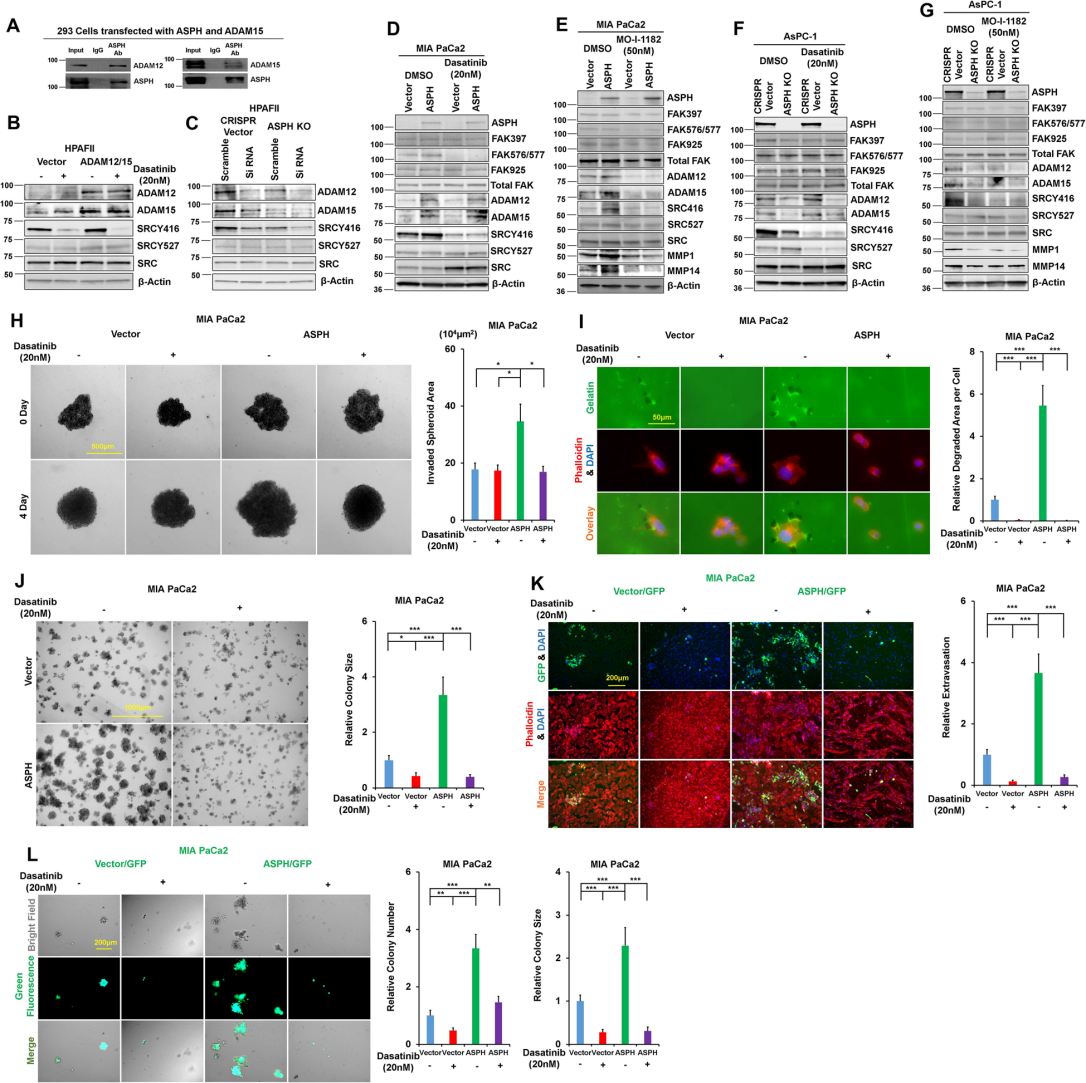
ASPH activates SRC signaling in pancreatic cancer. **a** A direct physical interaction of ASPH with ADAM12 or ADAM15 was detected by co-IP in HEK293 cells. **b** Endogenous ASPH-induced SRC activation (Y416) was efficiently inhibited by dasatinib. ADAM12 or ADAM15 overexpression substantially activated SRC was blocked by dasatinib. **c** ASPH KO or ADAM12/ADAM15 knock-down markedly downregulated SRC signal. **d**, **e** Exogeneous or **f**, **g** endogenous ASPH-mediated activation of SRC signaling was inhibited by both SMI and dasatinib. **h** 3-D tumor spheroid invasion in response to dasatinib. **i** ECM degradation/remodeling in response to dasatinib. **j** 3-D pancreatosphere formation in response to dasatinib. **k** Transendothelial migration and intravasation/extravasation. **l** Invasion through basement membrane and subsequent pancreatosphere formation cells in response to dasatinib. ^*^p < 0.05; ^**^p < 0.01; ^***^p < 0.001

Signaling pathways that act as downstream effectors of ASPH activity in pancreatic tumorigenesis are yet to be clarified. Based on bioinformatics, protein-protein interactions between ASPH and serial potential candidates were comprehensively screened. The ADAMs family members are critically involved in tumor pathogenesis. Accordingly, direct physical interactions of ASPH with ADAM12 and ADAM15 were identified by co-IP and Western blot (Fig. 2a). ADAM12 or ADAM15 interacts with SH3 domain of SRC [13–15], resulting in activation of the SRC cascade in pancreatic cancer cells (Fig. 2b). A high level of endogenous ASPH activated SRC (phosphorylated Y416), which was inhibited by Dasatinib (SRC inhibitor) in HPAFII. ADAM12/ADAM15 overexpression strengthened activation of SRC, which was blocked by Dasatinib (Fig. 2b). The ASPH KO, ADAM12, or ADAM15 knock-down (KD) prevented SRC from being activated in HPAFII (Fig. 2c). We hypothesized ASPH acts as an activator of SRC signaling to promote tumor progression in pancreatic cancer. Indeed, ASPH upregulated active form of SRC, which was impeded by both MO-I-1182 and Dasatinib (Fig. 2d, e). Then, we determined if inhibition of SRC activity can lessen ASPH-mediated pro-oncogenic properties. Notably, ASPH enhanced migration/invasion (Additional file 3: Figure S3B-C), 3-D invasion (Fig. 2h), ECM degradation/remodeling (Fig. 2i), stemness (Fig. 2j), and in vitro *metastasis* (Fig. 2k-l) were substantially diminished by Dasatinib in MIA-Paca2. Endogenous ASPH-induced SRC activation (Fig. 2f, g; Additional file 3: Figure S3A), migration/invasion (Additional file 3: Figure S3D-G), 3-D invasion (Additional file 3: Figure S3H), ECM degradation/remodeling (Additional file 3: Figure S3I-J), stemness (Additional file 3: Figure S3K-L), in vitro *metastasis* (Additional file 3: Figure S3M-N) were undermined by Dasatinib in AsPC-1 and HPAFII. Collectively, ASPH activated the SRC signaling pathway to generate and maintain malignant phenotypes in pancreatic cancer.

### ASPH-mediated aggressive malignant phenotypes stem from invadopodia-driven degrading/remodeling ECM

**Fig. 3:**
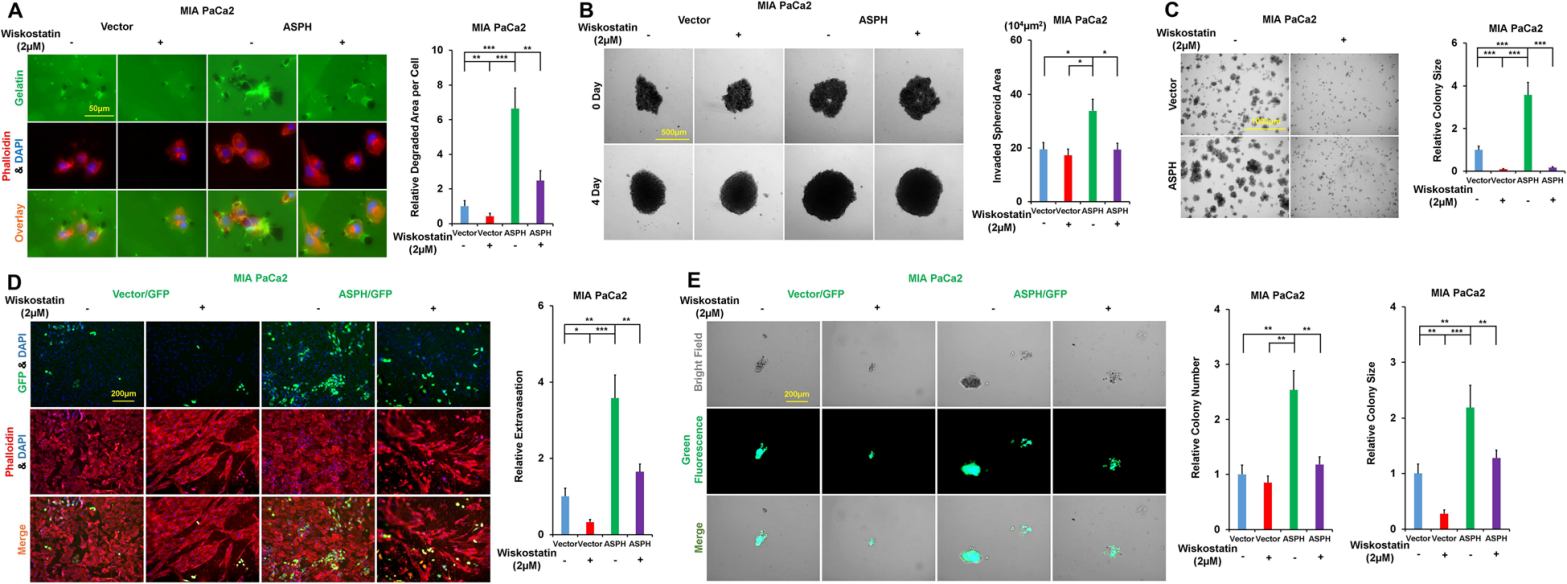
ASPH-SRC signal promotes invadopodia formation, maturation, and function in pancreatic cancer. **a** Invadopodia formation and ECM degradation/remodeling in response to Wiskostatin. **b** 3-D tumor spheroid invasion in response to Wiskostatin. **c** 3-D pancreatosphere formation in response to Wiskostatin. **d** Transendothelial migration and extravasation. **e** Invasion through basement membrane and subsequent pancreatosphere formation in response to Wiskostatin. ^*^p < 0.05; ^**^p < 0.01; ^***^p < 0.001

SRC signaling pathway is critical for invadopodia formation, maturation, and function [16]. Thus, we hypothesized the ASPH-SRC axis integrates invadopodia machinery to drive metastasis of pancreatic cancer cells. It has been reported that N-WASP enables MMP14 trafficking into invadopodia, provides the correct cytoskeletal framework to couple matrix remodeling with invadopodia [17], and assembles actin polymerization at invadopodia sites [18]. Then, could inhibition of N-WASP activity diminish ASPH-mediated pro-oncogenic properties? Exogenous ASPH enhanced malignant phenotypes, including migration/invasion (Additional file 4: Figure S4A), invadopodia formation-ECM degradation/remodeling (Fig. 3a), 3-D invasion (Fig. 3b), stemness (Fig. 3c), and in vitro metastasis (Fig 3d, e), were disassembled by N-WASP inhibitor Wiskostatin in MIA-Paca2. Endogenous ASPH-induced migration/invasion (Additional file 4: Figure S4B-C), invadopodia formation-ECM degradation/remodeling (Additional file 4: Figure S4D-E), 3-D invasion (Additional file 4: Figure S4F), stemness (Additional file 4: Fig. S4G-H), and in vitro metastasis (Additional file 4: Figure S4I-J) were deconstructed by Wiskostatin in AsPC-1 and HPAFII.

### ASPH promotes pancreatic cancer metastasis in vivo

**Fig. 4:**
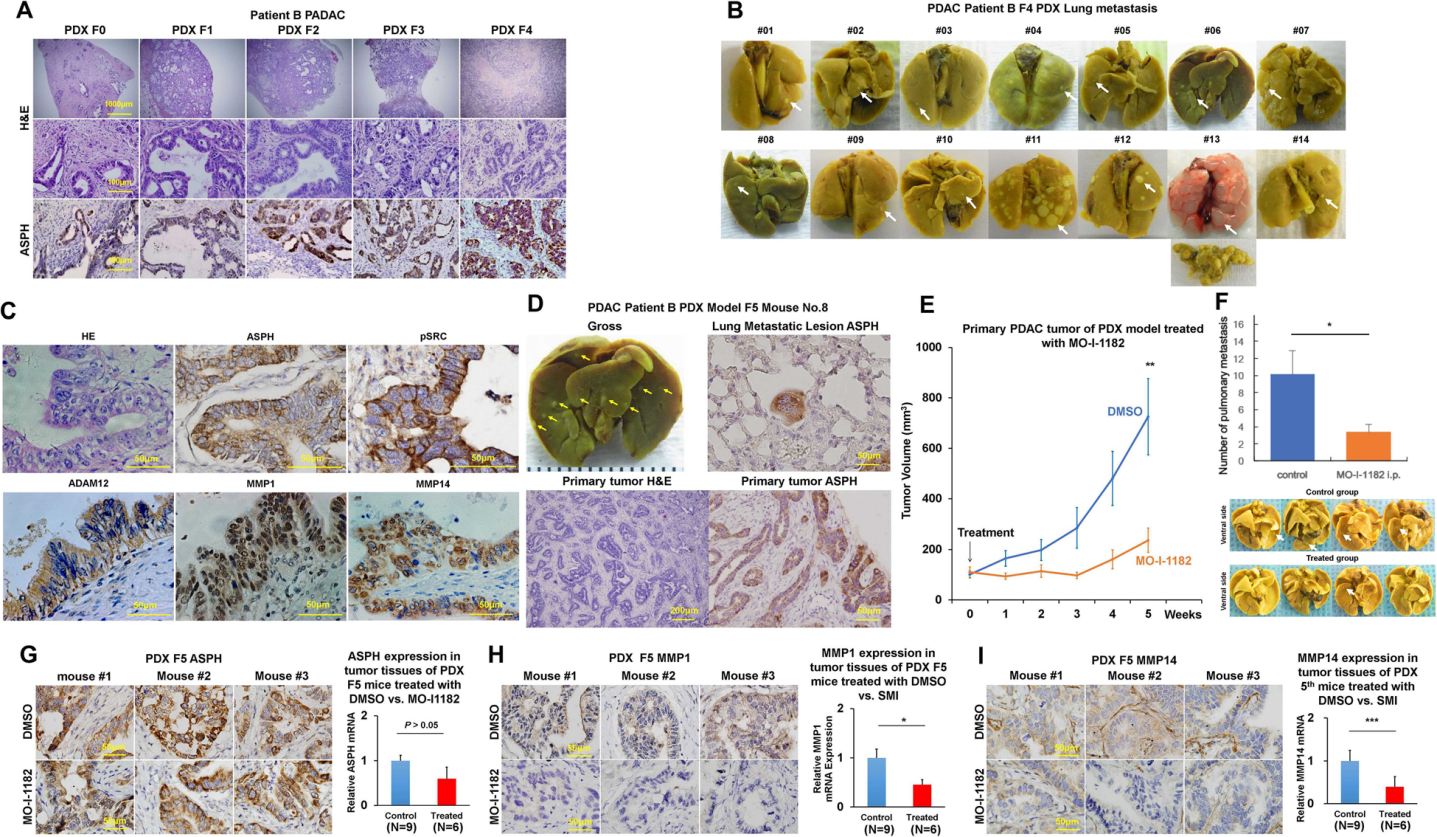
In vivo antitumor effects of a third-generation SMI (MO-I-1182) targeting ASPH enzymatic activity on PDX murine models of human PDAC. **a** Histopathologic characteristics (H&E) of original tumors (F0) derived from PDAC patient B and xenografted tumors in representative mice of F1 through F4 generation PDX model. **b** Pulmonary macro-metastases in representative F4 PDX mice derived from patient B. One hundred percent (14/14) of F4 PDX mice had spontaneously developed pulmonary metastasis. **c** Expression profiling of ASPH-SRC components in resected primary PDAC tumor specimens derived from patients B. **d** Gross appearance of the involved lungs, histopathologic characteristics, and expression profiling of ASPH in transplanted primary tumors as well as pulmonary macro-metastases in a representative mouse of the F5 generation PDX model derived from PDAC patient B. **e** Transplanted primary tumor growth in mice of the F5 generation PDX model in response to i.p. injected SMI vs. DMSO control. It took 4–5 weeks for the transplanted tumors to grow up to 100 mm^3^, when treatment with MO-I-1182 (10 mg/kg, i.p., every other day) was initiated. The mice were followed up for 5 weeks until the tumors grew up to 1000 mm^3^. **f** Antitumor effects of i.p. injected SMI on pulmonary metastasis. SMI blocks pulmonary micro-/macro-metastases in mice of F5 generation PDX derived from patient B. **g–i** Expression profiling of ASPH and MMPs detected by IHC and qRT-PCR in representative mice of the F5 generation PDX model treated with SMI vs. DMSO control. ^*^p < 0.05; ^**^p < 0.01; ^***^p < 0.001

Can ASPH-mediated pro-oncogenic properties be reversed/blocked by small molecule inhibitors in vivo? We explored the effects of MO-I-1182 on primary tumor growth and metastasis using a PDX (patient-derived xenograft) murine model of human PDAC. The demographic and pathologic characteristics of selected patients with PDAC are detailed in Additional file 7: Table S1. Three representative PDX tumors from F0 generation mice derived from three PDAC patients were serially propagated from F1 to F7 generation. Case #3 (patient B) had spontaneously developed pulmonary metastasis during disease progression whereas Case #1 (patient A) and Case #6 (patient C) were free from clinically detectable metastatic diseases (Additional file 7: Table S1). The histologic characteristics of the original PDAC tumors were compared with F1–F4 generation mice (Fig. 4a, b; Additional file 5: Figure S5A-B). The original histologic architecture was bona fide preserved in the PDX mice, where the transformed glandular epithelium was partially enclosed by a dense desmoplastic stroma. Glandular cellularity was gradually increased in the PDX following serial passage in the NSG mice. ASPH expression profiling in the original tumor derived from PDAC patients was continuously recapitulated following serial passages for over 56 weeks (Additional file 5: Figure S5C). Patient B with clinical pulmonary metastasis transmitted this phenotype faithfully to a subsequent PDX model from F1 to F7 generation mice (Fig. 4a; Additional file 5: Figure S5D-E). One hundred percent of PDX mice spontaneously developed macro-/micro-pulmonary metastases. ASPH expression was authentically maintained in the macro-/micro-pulmonary metastases (Fig. 4a–d). No pulmonary metastasis was observed in the PDX F1 to F7 generation mice derived from patients A and C.

### Small molecule inhibitor targeting ASPH inhibits primary tumor growth and pulmonary metastasis

To evaluate if ASPH activates SRC cascade in vivo, expression profiling of ASPH network was measured in the F4 to F7 generation PDX tumors derived from Case #3 (patient B) with transmissible spontaneous pulmonary metastasis, patients A and C without detectable metastasis. All three patients exhibited ASPH-positive PDX tumors through F4 to F7 generation. Only patient B displayed markedly activated SRC (pSRC Y416/418), upregulation of regulator ADAM12, and downstream MMPs (Fig. 4c). Patient B had the highest level of ASPH expression (in terms of immunostaining intensity [strong] and distribution [75–100%]) compared to patient A or C. Importantly, all the components in the ADAM12-SRC-MMPs axis were activated in patient B, but not in patient A or C. Tumor development/progression were accelerated with serial passage in the PDX model (Additional file 5: Figure S5F). The spontaneous pulmonary metastasis is proposed to be attributable to activation of SRC cascades in the primary PDX tumor. A SMI specifically targeting ASPH enzymatic activity exerted antitumor effects on PDAC growth and progression in vivo. F5 to F7 generation PDX tumors derived from patient B were allowed to grow subcutaneously for 4–5 weeks on the back of NSG mice. MO-I-1182 was administered i.p. daily for another 5 weeks. Primary PDX tumor growth (on the back) was monitored weekly. The animals were sacrificed 5 weeks later and the lungs examined for metastases. Primary PDAC tumor growth and pulmonary metastases were substantially inhibited with i.p. formulated MO-I-1182 preparation (Fig. 4e, f). SRC signaling components were consistently downregulated in response to ASPH enzymatic inhibition (Fig. 4g–i). Therefore, the small molecule inhibitor efficiently blocks PDAC development, progression, and metastatic spread to the lung.

### Expression profiling of the ASPH-SRC axis predicts clinical outcome of PDAC

**Fig. 5:**
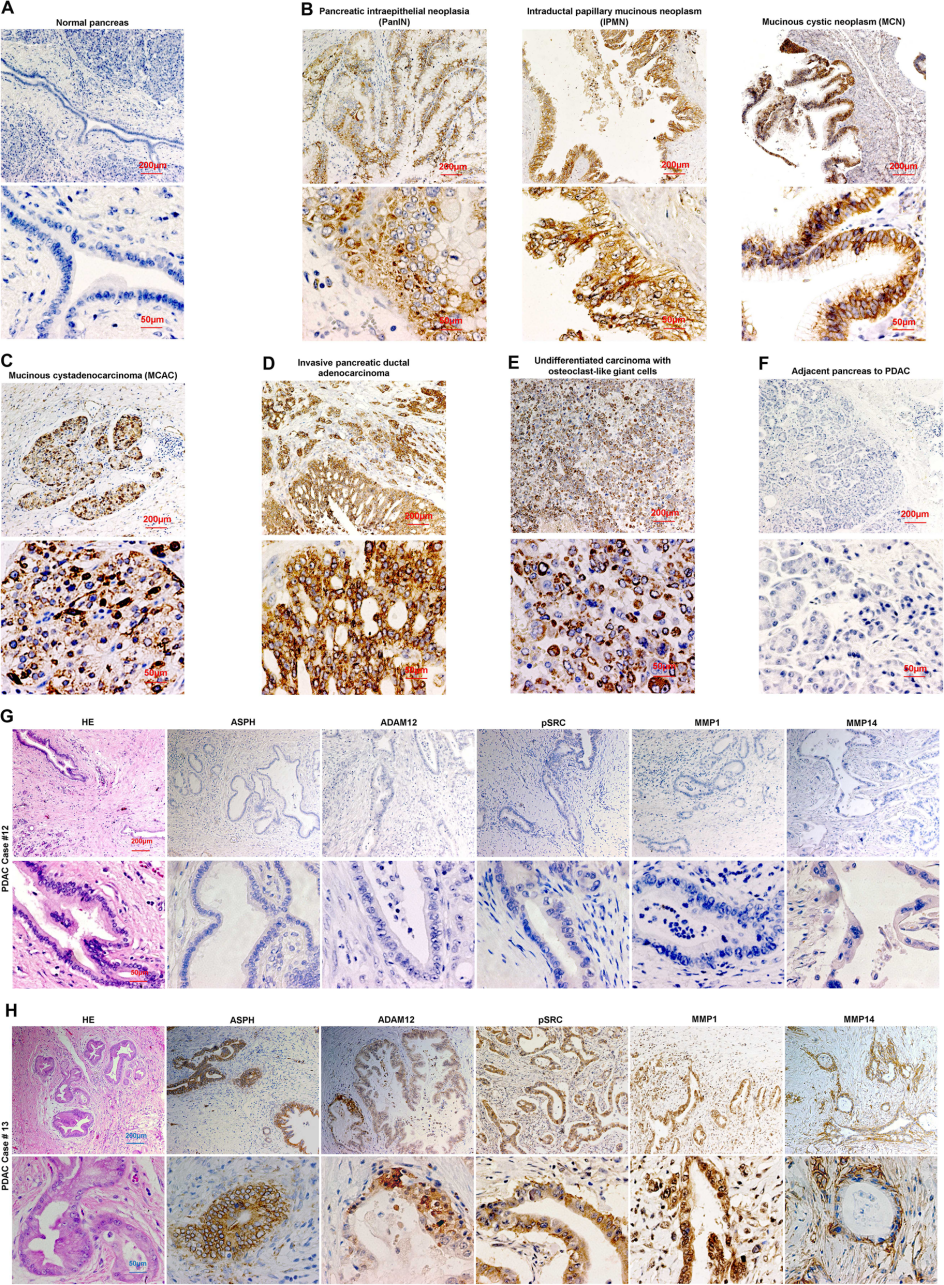
Expression profiling of ASPH network components in pancreatic cancer patients. **a–f** Expression of ASPH in **a** normal pancreas; **b** precursor lesions for sporadic pancreatic cancer: PanINs, IPMN, and MCN; **c** pancreatic MCAC; **d** invasive PDAC; **e** OGCs; **f** adjacent non-malignant pancreas. **g**, **h** Histopathologic characteristics and ASPH network expression profiling in two representative tumors derived from PDAC patients. SRC cascades were consistently **g** downregulated/silenced vs. **h** upregulated/activated in ASPH negative (patient #12) vs. positive (patient #13) PDAC

To confirm ASPH-SRC axis functions in PDAC patients, IHC was conducted to illustrate differential expression between tumor and adjacent non-malignant pancreatic tissue. The demographic/clinical features of the study population (*N* = 166) are summarized in Additional file 7: Table S2. The ASPH was detected in 97.6% of PDAC patients, with a negative, low, moderate, high, or very high expression rate of 2.4%, 22.9%, 23.5%, 27.7%, and 23.5%, respectively (Additional file 6: Figure S6A). ASPH was undetectable in adult normal pancreas (Fig. 5a), inflammatory diseases (acute/chronic pancreatitis), or pancreatic neuroendocrine tumor [12]. ASPH was upregulated at early stage preinvasive pancreatic neoplasm including pancreatic intraepithelial neoplasia (PanIN), intraductal papillary mucinous neoplasm (IPMN), and mucinous cystic neoplasm (MCN) (Fig. 5b), whereas it was markedly expressed in mucinous cystadenocarcinoma (MCAC) (Fig. 5c), invasively advanced/spontaneously metastatic pancreatic cancer (Fig. 5d), and extremely aggressive undifferentiated pancreatic carcinoma with osteoclast-like giant cells (UC-OGC) (Fig. 5e), compared to adjacent non-malignant pancreas (Fig. 5f).

**Fig. 6:**
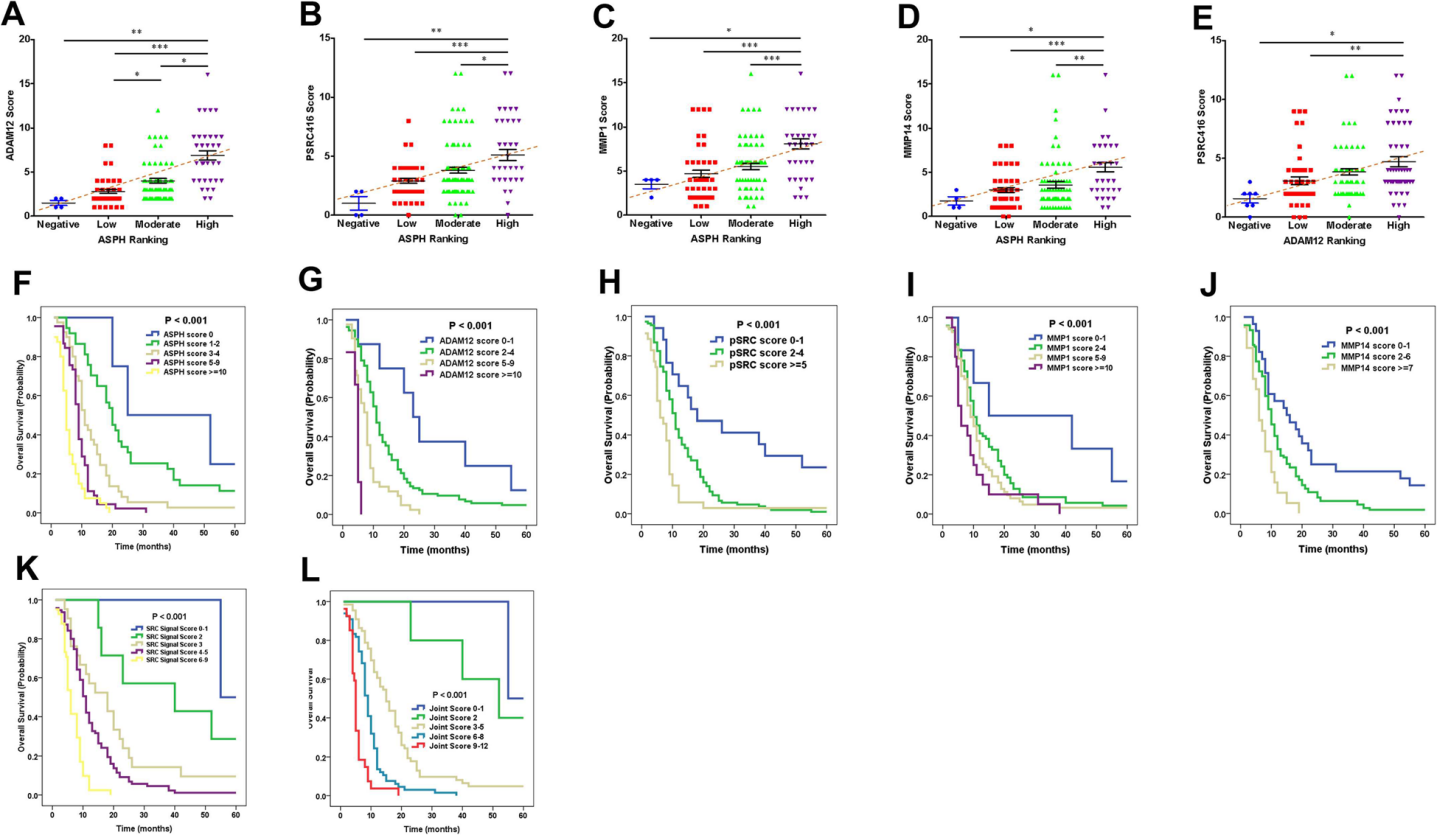
Expression levels of ASPH-Notch/SRC axis elements predict clinical outcome of PDAC patients. **a–e** ASPH expression level positively correlated with SRC components levels. Active SRC expression level positively correlated with ADAM12 level. Staining intensity and distribution of IHC were evaluated under × 10, × 40, × 100, and × 400 magnification. The average of percentage (number of positive staining cells/100 tumor cells) was calculated from at least 10 areas per high power field (HPF). **f–j** Compared to a negative-low level, a moderate-high level of ASPH; MMP1/14; ADAM12; or active SRC expression conferred reduced OS of PDAC patients (log-rank test, *p*s < 0.001). **k**, **l** Combined effects of four (SRC axis) or five (ASPH-SRC axis) molecules on OS of PDAC patients using the Kaplan-Meier method. The numbers from 0 to 12 indicate the total expression scores of at-risk proteins (log-rank test, *p* < 0.001). ^*^p < 0.05; ^**^p < 0.01; ^***^p < 0.001

SRC network components were consistently downregulated or upregulated in ASPH negative vs. positive PDAC patients. ASPH was moderately (very) strongly expressed in poorly differentiated more aggressively tumors, whereas it was negatively weakly expressed in moderately well differentiated less-invasive tumors (Fig. 5d, g, h; Additional file 6: Figure S6B-F). ADAM12-SRC signaling was activated in PDAC patients, where ASPH expression positively correlated with ADAM12 and active SRC (Fig. 6a–d). ADAM12 expression positively correlated with active SRC (Fig. 6e).

To explore if ASPH network level predicts clinical outcome of PDAC patients, Kaplan-Meier plot and Cox proportional hazards regression model were employed. Compared to a negative-low level, a moderate-very high level of ASPH, ADAM12, activated SRC, and MMPs correlated with curtailed overall survival (OS) of pancreatic cancer patients (log-rank test, *p*s < 0.001) (Fig. 6f–j).

Do ASPH network elements act synergistically on modulating clinical outcome of PDAC patients? A potential joint effect of individual molecules on OS was analyzed by combing the unfavorable expression scores significantly associated with reduced survival in the multivariate models. Increased numbers/expression levels of unfavorable molecules conferred reduced OS (Fig. 6k, l; Additional file 7: Table S3–4). Patients with 0–2 (*n* = 4), 3–5 (*n* = 8), 6–8 (*n* = 24), 9–12 (*n* = 73) of unfavorable expression scores of the five molecules had median survival time of 55.4, 15.9, 9.7, and 5.0 months, respectively (*p* < 0.001). Compared to patients with 0–2 unfavorable score, for those carrying 3–5, 6–8, or 9–12 unfavorable scores, adjusted hazard ratio (HR) (95% confidence interval) was 2.91 (0.59–14.40), 5.24 (1.22–22.49, 11.14 (2.60–47.81), and 18.30 (4.08–82.14), respectively (*p* < 0.001).

## Discussion

ASPH promotes pancreatic cancer progression through activating the SRC signaling pathway. The ASPH-SRC axis guides tumor cells to strengthen invadopodia formation/maturation and ECM degradation/remodeling, migration, invasion, stemness, transendothelial migration (intravasation/extravasation), and metastatic colonization/outgrowth at distant sites (e.g., lungs). In a dose-/intensity-dependent pattern, ASPH, active SRC, ADAM12, and MMPs independently and synergistically confer adverse prognosis of PDAC patients. Patient’s OS was curtailed with an increase in numbers/expression levels of unfavorable molecules. The ASPH-SRC axis components are proposed to be novel prognostic factors for PDAC.

ASPH mediates invadopodia formation/maturation/function, as described by enhanced degradation/remodeling of ECM and altered morphology in 3-D culture. As shown in Fig. 3d, N-WASP inhibitor Wiskostatin could not completely abolish the ASPH-mediated extravasation and invadopodia formation. Thus, ASPH could simultaneously depend on complex molecular mechanisms to maintain malignant phenotypes besides the N-WASP signal, which requires further investigation. ASPH activates SRC through directly interacting with ADAM12/ADAM15, which activates SRC [13–15]. SRC in turn enhances ADAM enzymatic activity [19–21] and forms a positive feedback loop. SRC pathway promotes angiogenesis, invadopodia formation [18, 22], and metastasis [23, 24]. Proteinase MMPs upregulated by the SRC signaling and ADAMs stabilized by ASPH are essential components for invadopodia architecture [18]. MMPs act as outlets of SRC cascade and direct executors for ECM degradation/remodeling to facilitate invasion and metastasis of pancreatic cancer cells.

The third-generation SMI will inhibit about 90–95% of ASPH’s enzymatic activity. Under this circumstance, ASPH’s enzymatic activity still remains at 5–10%. Therefore, MO-I-1182 does not abolish completely ASPH-mediated phenotypes.

It is challenging to develop and evaluate drugs targeting metastasis due to lack of suitable animal models that realistically mimics human tumor histopathology. We have established a novel PDX model of human PDAC that spontaneously metastasizes to murine lungs from a subcutaneous grown neoplasm on the back of NSG mice. This phenotype faithfully recapitulated histopathological/clinical characteristics of original tumor derived from specific patient and was serially propagated in 100% of the animals from the F2 to F7 generation, thus far. Therefore, ASPH is a potential driver of pulmonary metastasis where SRC signaling pathways are critically involved. ASPH activates SRC cascade in the primary PDAC tumor, upregulates downstream target genes (e.g., MMPs), and contributes to multiple steps of pancreatic cancer metastasis. Specific third-generation small molecule inhibitor targeting ASPH enzymatic activity substantially disrupted primary tumor growth and impeded pulmonary metastasis.

Metastasis is a complicated multi-step cascade conferring the deadly destination of cancer. Tumor cells disseminate from the primary site, intravasate into the vascular/lymphatic system, survive in circulation, extravasate across the endothelium, and eventually colonize secondary sites. However, how cancer cells intravasate/extravasate is yet to be illustrated. Invadopodia are cancer-specific protrusive and adhesive structures on the basolateral side of cultured cancer cells. However, physiological roles for invadopodia in cancer have not been established. Invadopodia concentrate proteases (MT1-MMP, MMP1) and Tks5 for local directed release and enzymatic activity for ECM degradation and remodeling [25, 26].

In randomly selected PDAC patients, expression levels of ASPH-SRC components independently predict prognosis of PDAC. The more harmful molecules patients harbor, the more deleterious clinical outcome is destinated. In adult normal pancreas, ASPH is silenced, in sharp contrast to be turned on during malignant transformation. ASPH is upregulated from pre-malignant pancreatic lesions (PanINs, MCN, and IPMN), substantially enhanced in MCAC, advanced/spontaneously metastatic pancreatic cancer, and extremely aggressive UC-OGC. SRC signaling elements were consistently downregulated or upregulated in ASPH negative vs. positive PDAC patients. Individual components of the ASPH-SRC axis vigorously and synergistically contribute to worse prognosis, dose- and intensity-dependently. More importantly, we have illustrated that ASPH can be measured by ELISA using patients’ serum samples. In this immunoassay, ASPH is detectable/significantly increased in cancer patients but undetectable or very tiny, if any, in health control donors. Therefore, ASPH is promising to serve as a biomarker for early diagnostics and prognostics of pancreatic cancer.

Limitations of this study include the following: (a) within ADAM12 or ADAM15, specific interaction/binding sites for and hydroxylation sites by ASPH have yet to be disclosed. Furthermore, upon hydroxylation by ASPH, dynamic configurational or functional changes of ADAM12/ADAM15 have yet to be explored. Whether this hydroxylation will be required for maintenance of SRC signal activation is under investigation. (b) To decipher prognostic values of ASPH network, a single-center-based retrospective study was designed, which may cause sampling bias. In addition, a limited number of proteins were investigated, and potential false-positive findings might be resulted from multiple comparisons [27, 28].

## Conclusions

Extensive efforts to characterize ASPH have revealed its key roles in multi-steps of pancreatic cancer metastasis: EMT, ECM degradation/remodeling, invasion through the basement membrane at the primary site, intravasation, survival in circulation, extravasation, and eventually metastatic colonization/outgrowth. Newly developed small molecule inhibitor bind to highly conserved catalytic site of ASPH, leading to substantial reduction in β-hydroxylase activity [6, 12, 29–31], abrogate downstream pro-oncogenic events induced by the SRC signaling pathway. Therefore, the ASPH-SRC axis-mediated pro-invasive invadopodia are collapsed following treatment and ASPH’s pro-metastatic properties are diminished in pancreatic cancer. Our study has provided direct evidence of functional roles for invadopodia during pancreatic cancer cell intravasation/extravasation and consequent distant metastasis, which has revealed an opportunity for optimal intervention in this clinically important process. This study together with previous findings [8, 9, 12, 30–40] establishes ASPH as a therapeutic target for pancreatic cancer. Importantly, we have demonstrated a paramount role of the ASPH-SRC axis in determining clinical outcome of patients with pancreatic cancer.

## Supplementary information

Additional file 1: **Figure S1.** ASPH mediated migration, invasion, EMT and ECM degradation/remodeling are reversed in vitro by a small molecule inhibitor (SMI) specifically against β-hydroxylase activity in PC. (A) Structure of candidate 3rd generation SMIs targeting ASPH enzymatic activity. (B-G) Migration/invasion index of (B-C) MIA Paca2 (expressing empty vector and ASPH, respectively); (D-E) AsPC-1 and (F-G) HPAFII (expressing CRISPR vector and ASPH KO, respectively) in response to SMI. (H-L) Expression of mesenchymal marker Vimentin (H-J) or epithelial marker E-cadherin (K-L) in response to SMI. (M) 3-D tumor spheroid invasion of AsPC-1 cells in response to SMI. (N-O) ECM degradation/remodeling of AsPC-1 and HPAFII cells in response to SMI. ^*^p<0.05; ^**^p<0.01; ^***^p<0.001.

Additional file 2: **Figure S2.** ASPH mediated cancer stemness are reversed in vitro by SMI specifically against β-hydroxylase activity in PC. (A) Expression of cancer stem cell marker CD44 in MIA Paca2 cells in response to SMI. (B) Expression of mesenchymal marker Vimentin or cancer stem cell marker CD44 in AsPC1 cells in response to SMI. (C-D) Expression of cancer stem cell marker EpCAM in AsPC1 cells in response to SMI. (E) Expression of cancer stem cell marker CD44 in AsPC1 cells in response to SMI. (F-G) Expression of cancer stem cell markers CD44 and EpCAM in HPAFII cells in response to SMI. (H-I) 3D pancreatosphere formation of AsPC-1 and HPAFII cells in response to SMI. (J) Transendothelial migration and intravasation/extravasation; (K) Invasion through basement membrane and subsequent pancreatosphere formation of AsPC-1 cells in response to SMI. ^*^p<0.05; ^**^p<0.01; ^***^p<0.001.

Additional file 3: **Figure S3.** ASPH activates SRC signaling pathways in PC. (A) ASPH enhanced activation of SRC signaling pathway in HPAFII cells, which was inhibited by both SMI and Dasatinib, but not DAPT. (B-G) Migration and Invasion index of MIA PaCa2, AsPC-1 or HPAFII cells in response to Dasatinib. (H) 3D tumor spheroid invasion of AsPC-1 cells in response to Dasatinib. (I-J) ECM degradation/remodeling in AsPC-1 and HPAFII cells in response to Dasatinib. (K-L) 3D Pancreatosphere formation of AsPC-1 and HPAFII cells in response to Dasatinib. (M) Transendothelial migration and intravasation/extravasation; (N) Invasion through basement membrane and subsequent pancreatosphere formation of AsPC-1 cells in response to Dasatinib. ^*^p<0.05; ^**^p<0.01; ^***^p<0.001.

Additional file 4: **Figure S4.** ASPH-SRC axis mediated aggressive malignant phenotypes in PC, which are significantly attenuated in vitro by N-WASP inhibitor Wiskostatin. (A-C) Migration/invasion index of PC cells in response to Wiskostatin. (D-E) Invadopodia formation and ECM degradation/remodeling in AsPC-1 and HPAFII cells in response to Wiskostatin. (F) 3D tumor spheroid invasion of AsPC-1 cells in response to Wiskostatin. (G-H) 3D pancreatosphere formation of AsPC-1 and HPAFII cells in response to Wiskostatin. (I) Transendothelial migration and extravasation; (J) Invasion through basement membrane and subsequent pancreatosphere formation of AsPC-1 cells in response to Wiskostatin. ^*^p<0.05; ^**^p<0.01; ^***^p<0.001.

Additional file 5: **Figure S5.** In vivo antitumor effects of SMI on PDAC PDX models. Expression profiling of ASPH network components in pancreatic cancer patients. (A) Expression profiling of ASPH in 6 surgically resected PADC tumors (Additional file 7: Table S3) as candidates for transplantation into the NSG mice for establishment of PDX models. Tumor specimens from Case#1, #2, #3, #6 were serially passaged to NSG mice. (B) Expression profiling of ASPH in original PDAC tumors from 3 representative patients (Patient A, Case#1; B, Case #3; C, Case #6) and a transplanted tumor in a representative mouse of F4 generation PDX model derived from Patient B. (C) Tumor growth in a representative F0 PDX mice derived from Patient A. (D) Pulmonary macro−/micro-metastases of a representative F2 PDX mouse derived from Patient B. (E) Gross appearance of the involved lungs, histopathologic characteristics (H&E) and expression profiling of ASPH in transplanted primary tumors as well as pulmonary macro-metastases in a representative mouse of F5 generation PDX model derived from PDAC Patient B. (F) Tumor development was accelerated with generation in PDX mice.

Additional file 6: **Figure S6.** Expression profiling of ASPH network components in PC patients. (A) A summary of ASPH immunoreactivity in tumorous tissue (compare to adjacent nonmalignant) derived from PDAC patients (N=166). (B) In primary tumor derived from a PDAC patient, SRC signaling pathway was inactive despite of (a negative-low expression of) ASPH due to lack of SRC expression. (C-F) Histopathological characteristics (H&E) and ASPH network components expression profiling of representative tumors derived from 4 PDAC patients. Consistent downregulation vs. upregulation of activated SRC (phosphorylated at Tyr416); ADAM12; MMP1; and MMP14 based on negative-low vs. moderate-high levels ASPH, compared to adjacent non-malignant pancreas tissues (*P*<0.001, 2-sided paired t test).

Additional file 7: **Table S1.** Demographic/clinical characteristics of patients and histopathological classification of PDAC tumors transplanted into the PDX model of NGS mice. Table S2 Patients' Characteristics (N=166). Table S3 Clinical Predictors for Overall Survival (N=166). Table S4 Molecular Predictors for Overall Survival (N=166).

## Abbreviations

2-D: Two-dimensional
3-D: Three-dimensional
ADAM: A Disintegrin and Metalloprotease Domain
ASPH: Aspartate β-hydroxylase
BSA: Bovine serum albumin
DLL: Delta-like
ECD: Extracellular domain
ECM: Extracellular matrix
EGF: Epidermal growth factor
EMT: Epithelial–mesenchymal transition
ERK: Extracellular signal-regulated kinase
FBS: Fetal bovine serum
FFPE: Formalin-fixed paraffin embedded
H&E: Hematoxylin and eosin
HCC: Hepatocellular carcinoma
HR: Hazard ratio
HUVEC: Human umbilical vein/vascular endothelium
I.P.: Intraperitoneal
IACUC: Institutional Animal Care and Use Committee
IHC: Immunohistochemistry
IPMN: Intraductal papillary mucinous neoplasm
KO: Knockout
MAPK: Mitogen-activated protein kinase
MCAC: Mucinous cystadenocarcinoma
MCN: Mucinous cystic neoplasm
MMP: Matrix metalloproteinase
NSG: NOD.Cg-PrkdcscidIl2rgtm1Wjl/SzJ; NOD-scid IL2Rgnull
N-WASP: Neural Wiskott-Aldrich syndrome protein
OS: Overall survival
PanIN: Pancreatic intraepithelial neoplasia
PDAC: Pancreatic ductal adenocarcinoma
PDX: Patient-derived xenograft
UC-OGC: Undifferentiated pancreatic carcinoma with osteoclast-like giant cells
