# Supplementary figures and images for: Aspartate β-hydroxylase promotes pancreatic ductal adenocarcinoma metastasis through activation of SRC signaling pathway
Source: J Hematol Oncol. 2019 Dec 30;12:144. doi: 10.1186/s13045-019-0837-z (PMC6937817; doi:10.1186/s13045-019-0837-z)

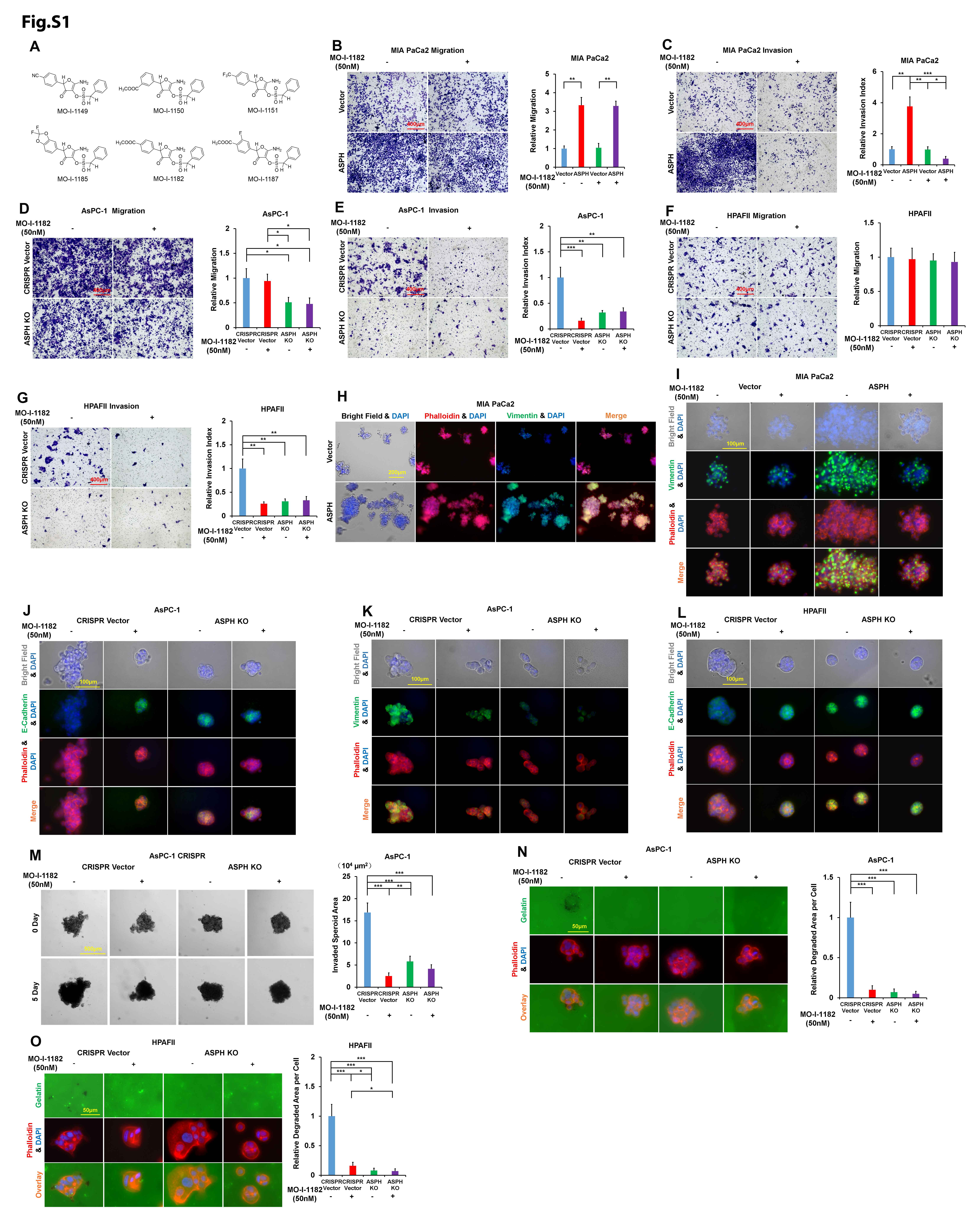

Supplement: Supplementary file 1 — Figure S1. ASPH mediated migration, invasion, EMT and ECM degradation/remodeling are reversed in vitro by a small molecule inhibitor (SMI) specifically against β-hydroxylase activity in PC. (A) Structure of candidate 3rd generation SMIs targeting ASPH enzymatic activity. (B-G) Migration/invasion index of (B-C) MIA Paca2 (expressing empty vector and ASPH, respectively); (D-E) AsPC-1 and (F-G) HPAFII (expressing CRISPR vector and ASPH KO, respectively) in response to SMI. (H-L) Expression of mesenchymal marker Vimentin (H-J) or epithelial marker E-cadherin (K-L) in response to SMI. (M) 3-D tumor spheroid invasion of AsPC-1 cells in response to SMI. (N-O) ECM degradation/remodeling of AsPC-1 and HPAFII cells in response to SMI. *p<0.05; **p<0.01; ***p<0.001. [file 13045_2019_837_MOESM1_ESM.jpg]

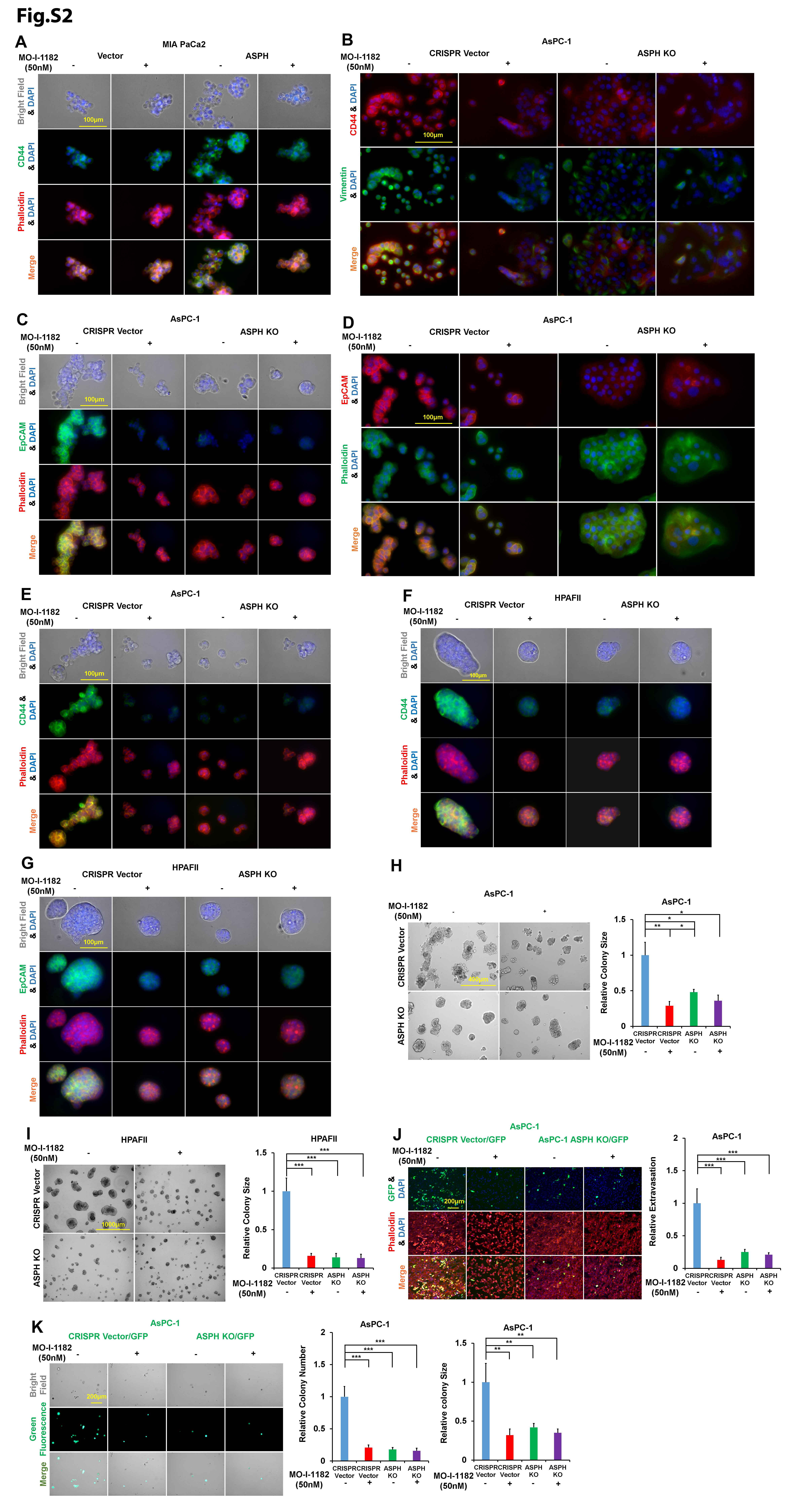

Supplement: Supplementary file 2 — Figure S2. ASPH mediated cancer stemness are reversed in vitro by SMI specifically against β-hydroxylase activity in PC. (A) Expression of cancer stem cell marker CD44 in MIA Paca2 cells in response to SMI. (B) Expression of mesenchymal marker Vimentin or cancer stem cell marker CD44 in AsPC1 cells in response to SMI. (C-D) Expression of cancer stem cell marker EpCAM in AsPC1 cells in response to SMI. (E) Expression of cancer stem cell marker CD44 in AsPC1 cells in response to SMI. (F-G) Expression of cancer stem cell markers CD44 and EpCAM in HPAFII cells in response to SMI. (H-I) 3D pancreatosphere formation of AsPC-1 and HPAFII cells in response to SMI. (J) Transendothelial migration and intravasation/extravasation; (K) Invasion through basement membrane and subsequent pancreatosphere formation of AsPC-1 cells in response to SMI. *p<0.05; **p<0.01; ***p<0.001. [file 13045_2019_837_MOESM2_ESM.jpg]

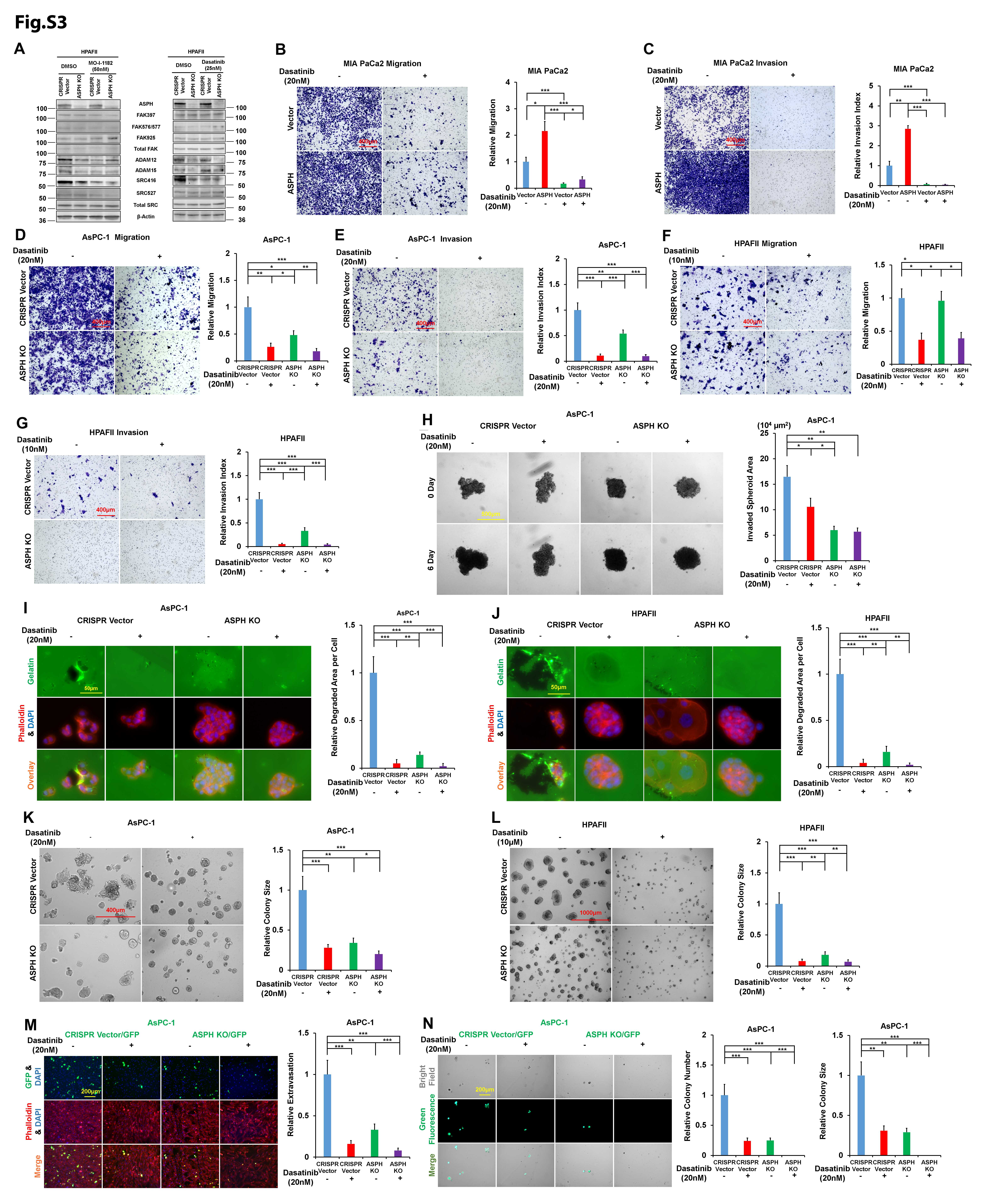

Supplement: Supplementary file 3 — Figure S3. ASPH activates SRC signaling pathways in PC. (A) ASPH enhanced activation of SRC signaling pathway in HPAFII cells, which was inhibited by both SMI and Dasatinib, but not DAPT. (B-G) Migration and Invasion index of MIA PaCa2, AsPC-1 or HPAFII cells in response to Dasatinib. (H) 3D tumor spheroid invasion of AsPC-1 cells in response to Dasatinib. (I-J) ECM degradation/remodeling in AsPC-1 and HPAFII cells in response to Dasatinib. (K-L) 3D Pancreatosphere formation of AsPC-1 and HPAFII cells in response to Dasatinib. (M) Transendothelial migration and intravasation/extravasation; (N) Invasion through basement membrane and subsequent pancreatosphere formation of AsPC-1 cells in response to Dasatinib. *p<0.05; **p<0.01; ***p<0.001. [file 13045_2019_837_MOESM3_ESM.jpg]

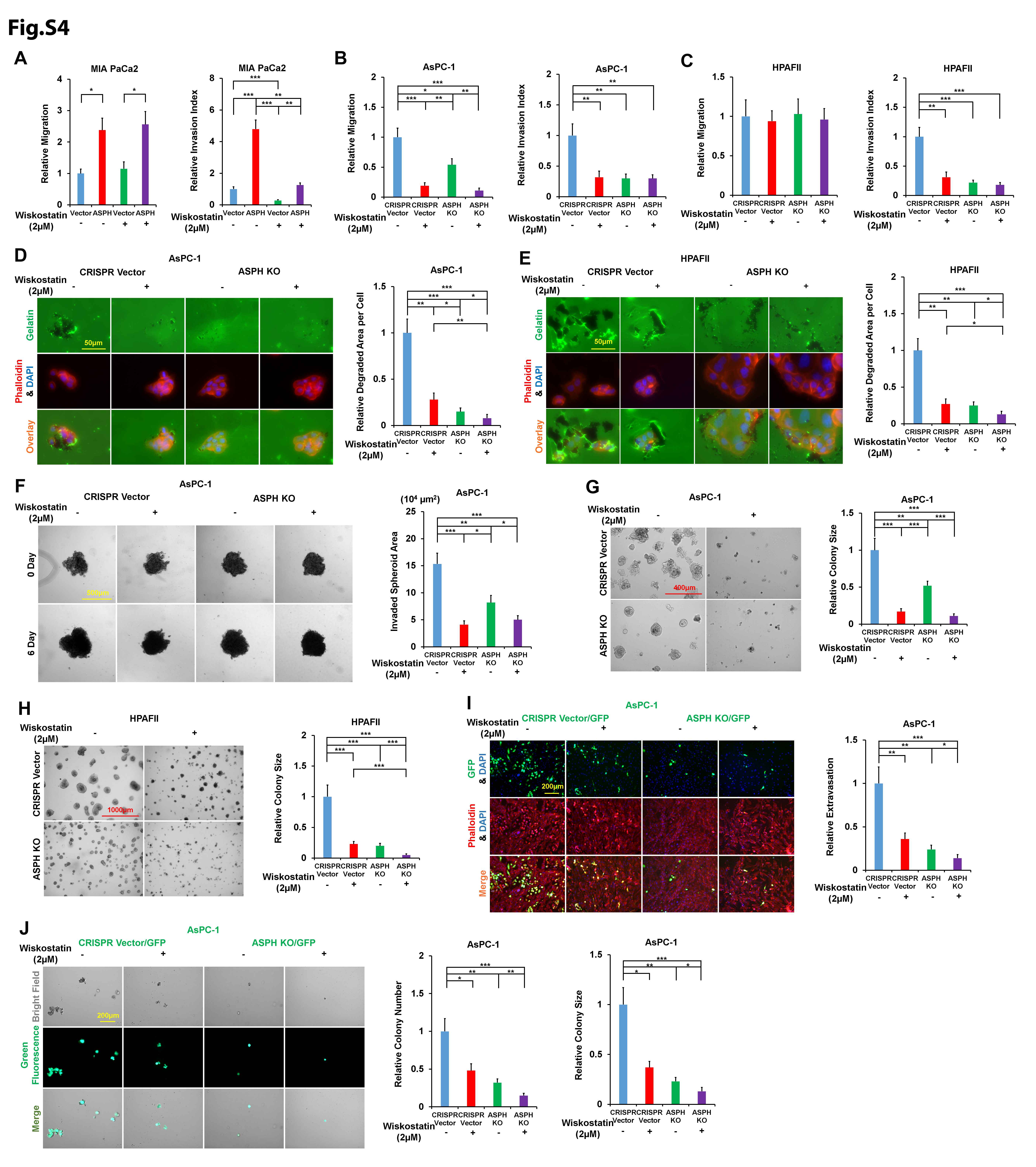

Supplement: Supplementary file 4 — Figure S4. ASPH-SRC axis mediated aggressive malignant phenotypes in PC, which are significantly attenuated in vitro by N-WASP inhibitor Wiskostatin. (A-C) Migration/invasion index of PC cells in response to Wiskostatin. (D-E) Invadopodia formation and ECM degradation/remodeling in AsPC-1 and HPAFII cells in response to Wiskostatin. (F) 3D tumor spheroid invasion of AsPC-1 cells in response to Wiskostatin. (G-H) 3D pancreatosphere formation of AsPC-1 and HPAFII cells in response to Wiskostatin. (I) Transendothelial migration and extravasation; (J) Invasion through basement membrane and subsequent pancreatosphere formation of AsPC-1 cells in response to Wiskostatin. *p<0.05; **p<0.01; ***p<0.001. [file 13045_2019_837_MOESM4_ESM.jpg]

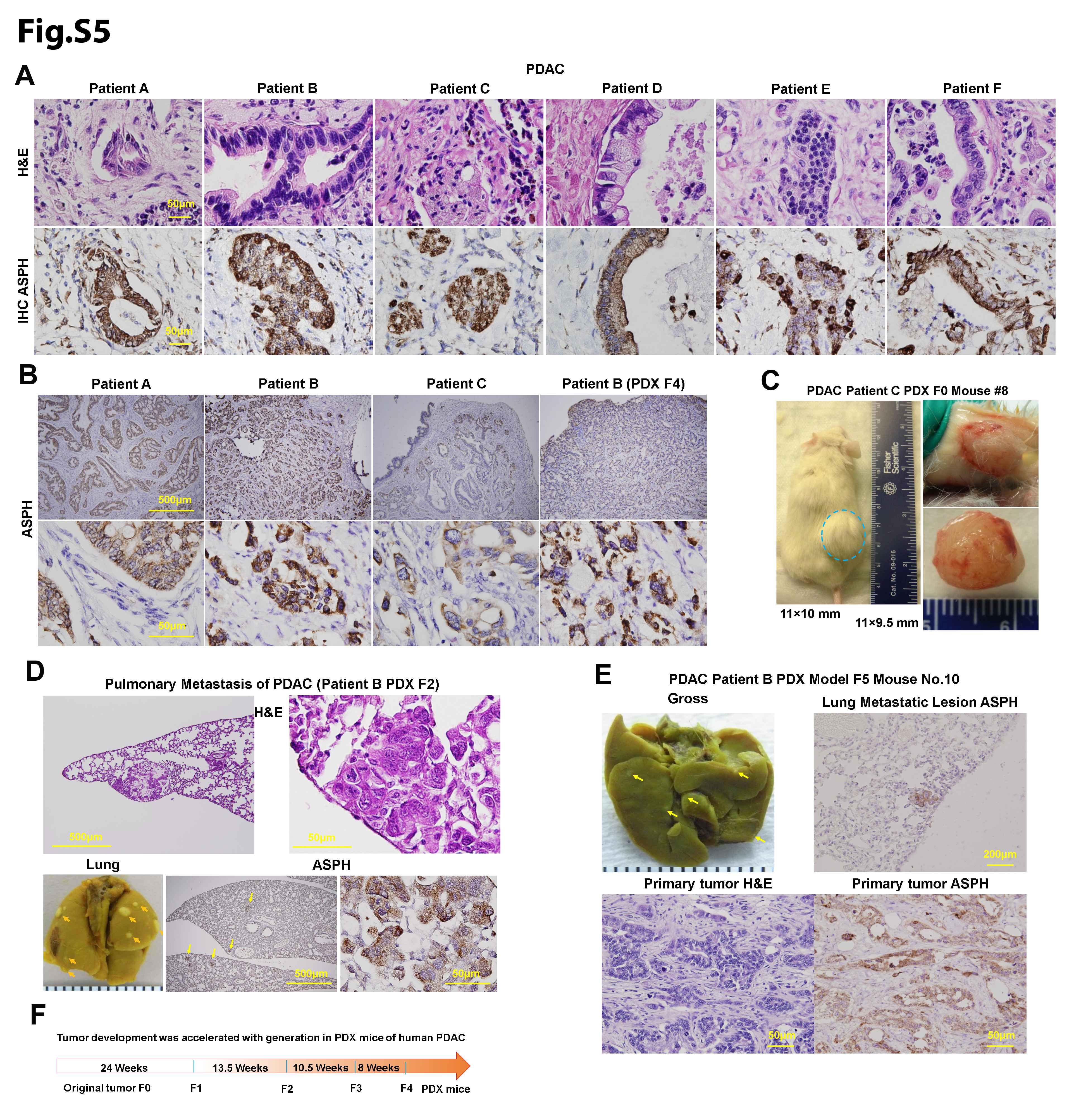

Supplement: Supplementary file 5 — Figure S5. In vivo antitumor effects of SMI on PDAC PDX models. Expression profiling of ASPH network components in pancreatic cancer patients. (A) Expression profiling of ASPH in 6 surgically resected PADC tumors (Additional file 7: Table S3) as candidates for transplantation into the NSG mice for establishment of PDX models. Tumor specimens from Case#1, #2, #3, #6 were serially passaged to NSG mice. (B) Expression profiling of ASPH in original PDAC tumors from 3 representative patients (Patient A, Case#1; B, Case #3; C, Case #6) and a transplanted tumor in a representative mouse of F4 generation PDX model derived from Patient B. (C) Tumor growth in a representative F0 PDX mice derived from Patient A. (D) Pulmonary macro−/micro-metastases of a representative F2 PDX mouse derived from Patient B. (E) Gross appearance of the involved lungs, histopathologic characteristics (H&E) and expression profiling of ASPH in transplanted primary tumors as well as pulmonary macro-metastases in a representative mouse of F5 generation PDX model derived from PDAC Patient B. (F) Tumor development was accelerated with generation in PDX mice. [file 13045_2019_837_MOESM5_ESM.jpg]

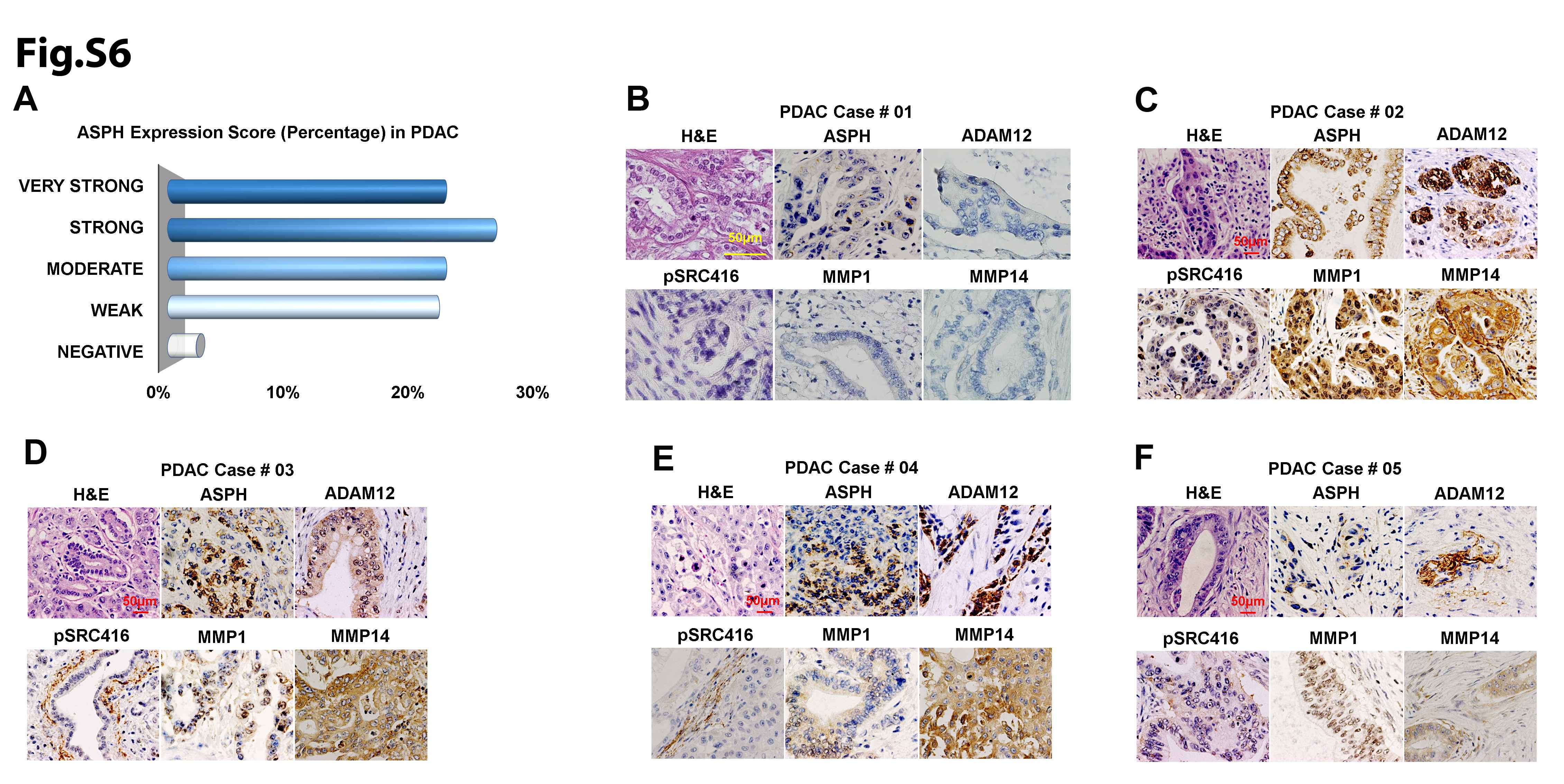

Supplement: Supplementary file 6 — Figure S6. Expression profiling of ASPH network components in PC patients. (A) A summary of ASPH immunoreactivity in tumorous tissue (compare to adjacent nonmalignant) derived from PDAC patients (N=166). (B) In primary tumor derived from a PDAC patient, SRC signaling pathway was inactive despite of (a negative-low expression of) ASPH due to lack of SRC expression. (C-F) Histopathological characteristics (H&E) and ASPH network components expression profiling of representative tumors derived from 4 PDAC patients. Consistent downregulation vs. upregulation of activated SRC (phosphorylated at Tyr416); ADAM12; MMP1; and MMP14 based on negative-low vs. moderate-high levels ASPH, compared to adjacent non-malignant pancreas tissues (P<0.001, 2-sided paired t test). [file 13045_2019_837_MOESM6_ESM.jpg]
