# Supplementary material for: Aspartate β-hydroxylase promotes pancreatic ductal adenocarcinoma metastasis through activation of SRC signaling pathway
Source: J Hematol Oncol. 2019 Dec 30;12:144. doi: 10.1186/s13045-019-0837-z (PMC6937817; doi:10.1186/s13045-019-0837-z)
Supplement: Supplementary file 7 — Table S1. Demographic/clinical characteristics of patients and histopathological classification of PDAC tumors transplanted into the PDX model of NGS mice. Table S2 Patients' Characteristics (N=166). Table S3 Clinical Predictors for Overall Survival (N=166). Table S4 Molecular Predictors for Overall Survival (N=166). [file 13045_2019_837_MOESM7_ESM.docx]

**Supplemental Figure Legends**

**Fig. S1** ASPH mediated migration, invasion, EMT and ECM degradation/remodeling are reversed *in vitro* by SMI specifically against β-hydroxylase activity in PC.

(**A**) Structure of candidate 3^rd^ generation SMIs targeting ASPH enzymatic activity.

(**B-G**) Migration/invasion index of (**B-C**) MIA Paca2 (expressing empty vector and ASPH, respectively); (**D-E**) AsPC-1 and (**F-G**) HPAFII (expressing CRISPR vector and ASPH KO, respectively) in response to SMI.

(**H-L**) Expression of mesenchymal marker Vimentin (**H-J**) or epithelial marker E-cadherin (**K-L**) in response to SMI.

(**M**) 3-D tumor spheroid invasion of AsPC-1 cells in response to SMI.

(**N-O**) ECM degradation/remodeling of AsPC-1 and HPAFII cells in response to SMI.

^*^*p*<0.05; ^**^*p*<0.01; ^***^*p*<0.001.

**Fig. S2** ASPH mediated cancer stemness are reversed *in vitro* by SMI specifically against β-hydroxylase activity in PC.

(**A**) Expression of cancer stem cell marker CD44 in MIA Paca2 cells in response to SMI.

(**B**) Expression of mesenchymal marker Vimentin or cancer stem cell marker CD44 in AsPC1 cells in response to SMI.

(**C-D**) Expression of cancer stem cell marker EpCAM in AsPC1 cells in response to SMI.

(**E**) Expression of cancer stem cell marker CD44 in AsPC1 cells in response to SMI.

(**F-G**) Expression of cancer stem cell markers CD44 and EpCAM in HPAFII cells in response to SMI.

**(H-I)** 3D pancreatosphere formation of AsPC-1 and HPAFII cells in response to SMI.

(**J**) Transendothelial migration and intravasation/extravasation; (**K**) Invasion through basement membrane and subsequent pancreatosphere formation of AsPC-1 cells in response to SMI.

^*^*p*<0.05; ^**^*p*<0.01; ^***^*p*<0.001.

**Fig. S3** ASPH activates SRC signaling pathways in PC.

(**A**) ASPH enhanced activation of SRC signaling pathway in HPAFII cells, which was inhibited by both SMI and Dasatinib, but not DAPT.

(**B-G**) Migration and Invasion index of MIA PaCa2, AsPC-1 or HPAFII cells in response to Dasatinib.

(**H**) 3D tumor spheroid invasion of AsPC-1 cells in response to Dasatinib.

(**I-J**) ECM degradation/remodeling in AsPC-1 and HPAFII cells in response to Dasatinib.

(**K-L**) 3D Pancreatosphere formation of AsPC-1 and HPAFII cells in response to Dasatinib.

(**M**) Transendothelial migration and intravasation/extravasation; (**N**) Invasion through basement membrane and subsequent pancreatosphere formation of AsPC-1 cells in response to Dasatinib.

^*^*p*<0.05; ^**^*p*<0.01; ^***^*p*<0.001.

**Fig. S4** ASPH-SRC axis mediated aggressive malignant phenotypes in PC, which are significantly attenuated *in vitro* by N-WASP inhibitor Wiskostatin.

(**A-C**) Migration/invasion index of PC cells in response to Wiskostatin.

(**D-E**) Invadopodia formation and ECM degradation/remodeling in AsPC-1 and HPAFII cells in response to Wiskostatin.

(**F**) 3D tumor spheroid invasion of AsPC-1 cells in response to Wiskostatin.

(**G-H**) 3D pancreatosphere formation of AsPC-1 and HPAFII cells in response to Wiskostatin.

(**I**) Transendothelial migration and extravasation; (**J**) Invasion through basement membrane and subsequent pancreatosphere formation of AsPC-1 cells in response to Wiskostatin.

^*^*p*<0.05; ^**^*p*<0.01; ^***^*p*<0.001.

**Fig. S5** *In vivo* antitumor effects of SMI on PDAC PDX models. Expression profiling of ASPH network components in pancreatic cancer patients.

(**A**) Expression profiling of ASPH in 6 surgically resected PADC tumors (**Supplemental Table 3**) as candidates for transplantation into the NSG mice for establishment of PDX models. Tumor specimens from Case#1, #2, #3, #6 were serially passaged to NSG mice.

(**B**) Expression profiling of ASPH in original PDAC tumors from 3 representative patients (Patient A, Case#1; B, Case #3; C, Case #6) and a transplanted tumor in a representative mouse of F4 generation PDX model derived from Patient B.

(**C**) Tumor growth in a representative F0 PDX mice derived from Patient A.

(**D**) Pulmonary macro-/micro-metastases of a representative F2 PDX mouse derived from Patient B.

(**E**) Gross appearance of the involved lungs, histopathologic characteristics (H&E) and expression profiling of ASPH in transplanted primary tumors as well as pulmonary macro-metastases in a representative mouse of F5 generation PDX model derived from PDAC Patient B.

(**F**) Tumor development was accelerated with generation in PDX mice.

**Fig. S6** Expression profiling of ASPH network components in PC patients.

(**A**) A summary of ASPH immunoreactivity in tumorous tissue (compare to adjacent nonmalignant) derived from PDAC patients (N=166).

(**B**) In primary tumor derived from a PDAC patient, SRC signaling pathway was inactive despite of (a negative-low expression of) ASPH due to lack of SRC expression.

(**C-F**) Histopathological characteristics (H&E) and ASPH network components expression profiling of representative tumors derived from 4 PDAC patients. Consistent downregulation vs. upregulation of activated SRC (phosphorylated at Tyr416); ADAM12; MMP1; and MMP14 based on negative-low vs. moderate-high levels ASPH, compared to adjacent non-malignant pancreas tissues (*P*<0.001, 2-sided paired t test).

**Supplementary** **Tables**

**Table S1. Demographic/clinical characteristics of patients and histopathological classification of PDAC tumors transplanted into the PDX model of NGS mice**

| Patient ID | Gender | Age | Location of  PDAC | T | N | AJCC  stage | Histopathology Grade/Differentiation |
| --- | --- | --- | --- | --- | --- | --- | --- |
| *1 (A) | F | 54 | HEAD | T3 | N1 | IIB | G2/Moderate |
| 2 | F | 35 | HEAD | T2 | N0 | IB | G1/Well |
| ^¶^3 (B) | F | 79 | TAIL | T3 | N1 | IIB | G3/Poor |
| 4 | M | 83 | HEAD | T3 | N0 | IIA | G3/Poor |
| 5 | M | 67 | HEAD | T3 | N1 | IIB | G1/Well |
| *6 (C) | F | 64 | HEAD | T3 | N1 | IIB | G3/Poor |

*No metastatic disease; ^¶^Pulmonary metastasis

**Table S2. Patients' Characteristics (N=166)**

| **Variable** | **No. of patients (%)** |
| --- | --- |
| Gender | |
| Female | 59 (35.5) |
| Male | 107 (64.5) |
| Age (y) | |
| ≤50 | 46 (27.7) |
| 51–60 | 55 (33.1) |
| 61–70 | 51 (30.7) |
| >70 | 14 (8.4) |
| CA 19-9 (U/mL) | |
| ≤37 | 46 (27.7) |
| >37 | 120 (72.3) |
| CEA (U/mL) | |
| ≤3 | 64 (46.7) |
| >3 | 73 (53.3) |
| Tumor site | |
| Head | 119 (71.7) |
| Body | 34 (20.5) |
| Tail | 13 (10.9) |
| Tumor size (cm) | |
| <4 | 38 (22.9) |
| ≥4 | 128 (77.1) |
| T stage |  |
| Tis, T1 | 141 (86.0) |
| T2-T4 | 23 (14.0) |
| Lymph node stage | |
| No | 125 (75.8) |
| Yes | 40 (24.2) |
| Micro-metastasis | |
| No | 147 (89.1) |
| Yes | 18 (10.9) |
| TNM stage |  |
| 0 | 88 (53.0) |
| I | 32 (19.3) |
| II | 29 (17.5) |
| III+IV | 17 (10.2) |
| Tumor differentiation | |
| Well | 8 (4.8) |
| Moderately | 102 (61.4) |
| Poorly | 56 (33.7) |
| Resection margin | |
| Negative (R0) | 151 (91.5) |
| Positive (R1) | 14 (8.5) |
| Anastomosis |  |
| Yes | 112 (76.2) |
| No | 35 (23.8) |
| Biochemical index ^a^ | |
| 0 | 21 (12.7) |
| 1-3 | 87 (52.4) |
| ≥4 | 58 (34.9) |

^a^ Biochemical index represents the number of serum markers with abnormal value, including aspartate aminotransferase, alanine aminotransferase, albumin, and bilirubin.

**Table S3. Clinical Predictors for Overall Survival (N=166)**

| **Variable** | **No. of patients** | **No. of deaths** | **MST (months)** | **P (log-rank)** | **HR (95% CI)** | **P^¶^** |
| --- | --- | --- | --- | --- | --- | --- |
| CA 19-9 (U/mL) | |  |  | 0.002 |  |  |
| ≤37 | 46 | 41 | 11.7 |  | 1.0 (Ref.) |  |
| >37 | 120 | 117 | 9.1 |  | 1.77 (1.23-2.55) | 0.002 |
| CEA (U/mL) | |  |  | 0.03 |  |  |
| ≤3 | 64 | 63 | 12.8 |  | 1.0 (Ref.) |  |
| >3 | 73 | 70 | 9.6 |  | 1.52 (1.10-2.11) | 0.01 |
| Tumor site | |  |  | 0.303 |  |  |
| Head | 119 | 111 | 10.7 |  | 1.0 (Ref.) |  |
| Body | 34 | 32 | 10.1 |  | 0.98 (0.66-1.46) | 0.93 |
| Tail | 13 | 13 | 9.7 |  | 1.58 (0.85-2.93) | 0.15 |
| Tumor size (cm) | | |  | 0.02 |  |  |
| <4 | 38 | 36 | 15.4 |  | 1.0 (Ref.) |  |
| ≥4 | 128 | 122 | 9.6 |  | 1.55 (1.06-2.28) | 0.02 |
| T stage |  |  |  | <0.001 |  |  |
| Tis, T1 | 141 | 134 | 10.8 |  | 1.0 (Ref.) |  |
| T2-T4 | 23 | 23 | 6.2 |  | 2.41 (1.52-3.82) | <0.001 |
| Lymph node stage | |  |  | 0.593 |  |  |
| No | 125 | 117 | 10.9 |  | 1.0 (Ref.) |  |
| Yes | 40 | 40 | 10.1 |  | 1.11 (0.77-1.60) | 0.57 |
| Micro-metastasis | |  |  | 0.019 |  |  |
| No | 147 | 139 | 10.8 |  | 1.0 (Ref.) |  |
| Yes | 18 | 18 | 7.0 |  | 1.78 (1.08-2.93) | 0.02 |
| TNM stage |  |  |  | 0.03 |  |  |
| 0 | 88 | 82 | 11.8 |  | 1.0 (Ref.) |  |
| I-IV | 78 | 78 | 9.0 |  | 1.41 (1.02-1.94) | 0.038 |
| Tumor differentiation | |  |  | 0.633 |  |  |
| Well | 8 | 8 | 10.9 |  | 1.0 (Ref.) |  |
| Moderately | 102 | 95 | 10.0 |  | 1.27 (0.61-2.64) | 0.53 |
| Poorly | 56 | 55 | 9.7 |  | 1.35 (0.63-2.89) | 0.44 |
| Resection margin | | | | 0.631 |  |  |
| (-) (R0) | 151 | 143 | 10.6 |  | 1.0 (Ref.) |  |
| (+) (R1) | 14 | 14 | 8.8 |  | 1.17 (0.66-2.08) | 0.58 |
| Anastomosis | |  |  | 0.03 |  |  |
| Yes | 112 | 108 | 11.6 |  | 1.0 (Ref.) |  |
| No | 35 | 34 | 8.1 |  | 1.69 (1.11-2.57) | 0.015 |
| Biochemical index ^a^ | | |  | 0.001 |  |  |
| 0 | 21 | 18 | 19.8 |  | 1.0 (Ref.) |  |
| 1--3 | 87 | 85 | 10.3 |  | 2.30 (1.33-3.99) | 0.003 |
| ≥4 | 58 | 57 | 8.1 |  | 2.75 (1.55-4.88) | <0.001 |

^a^ Biochemical index represents the number of serum markers with abnormal value, including aspartate aminotransferase, alanine aminotransferase, albumin, and bilirubin.

P^¶^ was from multivariate Cox proportional hazards regression model adjusted by gender and age.

**Table S4. Molecular Predictors for Overall Survival (N=166)**

| **Variable** | **No. of patients** | **No. of deaths** | **MST (months)** | **P (log-rank)** | **HR (95% CI)** | **P^¶^** |
| --- | --- | --- | --- | --- | --- | --- |
| ASPH expression score | | |  | <0.001 |  |  |
| 0-2 | 41 | 35 | 20.9 |  | 1.0 (Ref.) |  |
| 3-4 | 39 | 37 | 11.7 |  | 2.38 (1.47-3.84) | <0.001 |
| 5-9 | 46 | 46 | 9.1 |  | 4.51 (2.77-7.35) | <0.001 |
| ≥10 | 40 | 40 | 5.0 |  | 8.62 (5.15-14.43) | <0.001 |
| Active SRC expression score | | |  | <0.001 |  |  |
| 0-1 | 17 | 13 | 18.7 |  | 1.0 (Ref.) |  |
| 2-4 | 114 | 111 | 11.2 |  | 2.77 (1.50-5.09) | <0.001 |
| ≥5 | 35 | 34 | 6.4 |  | 5.86 (2.89-11.88) | <0.001 |
| ADAM12 expression score | | |  | <0.001 |  |  |
| 0-1 | 8 | 7 | 23.9 |  | 1.0 (Ref.) |  |
| 2-4 | 110 | 102 | 11.5 |  | 2.22 (1.02-4.84) | 0.045 |
| 5-9 | 42 | 42 | 8.3 |  | 5.08 (2.19-11.78) | <0.001 |
| ≥10 | 6 | 6 | 5.1 |  | 17.67 (5.47-57.09) | <0.001 |
| MMP1 expression score | | |  | 0.01 |  |  |
| 0-1 | 6 | 5 | 15.8 |  | 1.0 (Ref.) |  |
| 2-4 | 73 | 69 | 10.9 |  | 2.18 (0.87-5.47) | 0.097 |
| 5-9 | 67 | 64 | 9.5 |  | 3.13 (1.19-8.22) | 0.02 |
| ≥10 | 20 | 20 | 6.0 |  | 3.76 (1.38-10.30) | 0.01 |
| MMP14 expression score | | |  | <0.001 |  |  |
| 0-1 | 28 | 24 | 15.8 |  | 1 (Ref.) |  |
| 2-6 | 119 | 115 | 10.2 |  | 2.01 (1.26-3.20) | 0.003 |
| ≥7 | 19 | 19 | 5.9 |  | 3.99 (2.08-7.65) | <0.001 |
| Joint score for 5-Gene expression | |  |  | <0.001 |  |  |
| 0-2 | 7 | 4 | 55.4 |  | 1.0 (Ref.) |  |
| 3-5 | 66 | 61 | 15.9 |  | 4.85 (1.72-13.67) | 0.003 |
| 6-8 | 66 | 66 | 9.7 |  | 12.35 (4.28-35.65) | <0.001 |
| 9-12 | 27 | 27 | 5.0 |  | 31.40 (10.29-95.84) | <0.001 |

P^¶^ was from multivariate Cox proportional hazards regression model adjusted by gender, age, CA19-9, CEA, tumor size, tumor site, TNM stage, tumor differentiation, resection margin, anastomosis and Biochemical index.
